# Raman spectroscopic analysis of skin as a diagnostic tool for Human African Trypanosomiasis

**DOI:** 10.1371/journal.ppat.1010060

**Published:** 2021-11-15

**Authors:** Alexandre Girard, Anneli Cooper, Samuel Mabbott, Barbara Bradley, Steven Asiala, Lauren Jamieson, Caroline Clucas, Paul Capewell, Francesco Marchesi, Matthew P. Gibbins, Franziska Hentzschel, Matthias Marti, Juan F. Quintana, Paul Garside, Karen Faulds, Annette MacLeod, Duncan Graham

**Affiliations:** 1 Wellcome Centre for Integrative Parasitology, Institute of Biodiversity Animal Health and Comparative Medicine, University of Glasgow, Glasgow, United Kingdom; 2 Department of Biomedical Engineering, Texas A&M University, College Station, Texas, United States of America; 3 Center for Remote Health Technologies and Systems, Texas A&M Engineering Experimentation Station, College Station, Texas, United States of America; 4 Department of Pure and Applied Chemistry, University of Strathclyde, Glasgow, United Kingdom; 5 Veterinary Diagnostic Services, School of Veterinary Medicine, University of Glasgow, Glasgow, United Kingdom; 6 Wellcome Centre for Integrative Parasitology, Institute of Infection, Immunity and Inflammation, University of Glasgow, Glasgow, United Kingdom; 7 Institute of Infection, Immunity and Inflammation, University of Glasgow, Glasgow, United Kingdom; University Wuerzburg, GERMANY

## Abstract

Human African Trypanosomiasis (HAT) has been responsible for several deadly epidemics throughout the 20th century, but a renewed commitment to disease control has significantly reduced new cases and motivated a target for the elimination of *Trypanosoma brucei gambiense*-HAT by 2030. However, the recent identification of latent human infections, and the detection of trypanosomes in extravascular tissues hidden from current diagnostic tools, such as the skin, has added new complexity to identifying infected individuals. New and improved diagnostic tests to detect *Trypanosoma brucei* infection by interrogating the skin are therefore needed. Recent advances have improved the cost, sensitivity and portability of Raman spectroscopy technology for non-invasive medical diagnostics, making it an attractive tool for *gambiense*-HAT detection. The aim of this work was to assess and develop a new non-invasive diagnostic method for *T*. *brucei* through Raman spectroscopy of the skin. Infections were performed in an established murine disease model using the animal-infective *Trypanosoma brucei brucei* subspecies. The skin of infected and matched control mice was scrutinized *ex vivo* using a confocal Raman microscope with 532 nm excitation and *in situ* at 785 nm excitation with a portable field-compatible instrument. Spectral evaluation and Principal Component Analysis confirmed discrimination of *T*. *brucei-*infected from uninfected tissue, and a characterisation of biochemical changes in lipids and proteins in parasite-infected skin indicated by prominent Raman peak intensities was performed. This study is the first to demonstrate the application of Raman spectroscopy for the detection of *T*. *brucei* by targeting the skin of the host. The technique has significant potential to discriminate between infected and non-infected tissue and could represent a unique, non-invasive diagnostic tool in the goal for elimination of *gambiense*-HAT as well as for Animal African Trypanosomiasis (AAT).

## Author summary

Human African Trypanosomiasis (HAT), also known as sleeping sickness, is a disease caused by the parasite *Trypanosoma brucei* and has been responsible for the death of millions of people across Africa in the 20^th^ century. It is also a major economic burden for countries endemic for trypanosomiasis, affecting livestock productivity in rural areas (Animal African Trypanosomiasis). A long-term international collaboration with the help of the World Health Organisation has resulted in the rate of human infection decreasing to less than 1000 new cases per year. However, the human disease continues to spread within remote villages. Current diagnosis is based on the detection of parasites in blood and serum samples, but this is challenging during chronic human infections with low or non-detectable parasitaemia. However, the recent discovery of extravascular skin-dwelling trypanosomes indicates that a reservoir of infection remains undetected, threatening the effort to eliminate the disease. In this study we have targeted the skin as a site for diagnosis using Raman spectroscopy and demonstrate that this method showed great promise in the laboratory, laying the foundation for field studies to examine its potential to strengthen current diagnostic strategies for detecting HAT cases.

## Introduction

Human African Trypanosomiasis (HAT) or sleeping sickness is a neglected tropical disease present in sub-Saharan Africa and transmitted by tsetse flies. The disease is caused by the parasites *Trypanosoma brucei (T*. *b*.*) rhodesiense* and *T*. *b*. *gambiense*, which present as acute and chronic disease respectively. A third sub-species of this parasite, *T*. *b*. *brucei* is not human-infective but infects a wide range of domestic and game animals. These trypanosome parasites are extracellular and live within the blood and tissue fluids of their host, causing a wide spectrum of clinical symptoms, such as headache and fever, that are often mistaken for malaria [1], and eventually crossing the blood-brain barrier leading to neurological symptoms and significant morbidity and mortality if left untreated [1].

*Gambiense*-HAT, the most common form of the disease [2], has been targeted for elimination by the WHO, with the goal of interrupting disease transmission by 2030 [3,4]. As a result of concerted control measures, the number of reported annual cases has decreased from 40,000 in 1998 to 997 in 2018, although the estimated true number of cases is much higher [5,6]. Implementation of control, detection, and treatment is challenged by the rural and remote location of the communities most affected by HAT, which are difficult to access by medical teams, and many cases remain overlooked. As we approach the elimination goal for *T*. *b*. *gambiense* we must now focus on identifying and treating the last cases and carriers of disease to safeguard against a resurgence. Attention has turned to the development of innovative diagnostic measures (Rapid Diagnostic Tests, trypanolysis) and safer medicines that will support sustainable elimination and facilitate case detection and treatment in persistent disease foci [7–10].

In the absence of a vaccine, disease control relies on active case detection and treatment. Diagnosis centres rely on the large scale screening of at-risk populations using the card agglutination test for trypanosomiasis (CATT), developed in the late 1970s [11], supported by the recent introduction of individual lateral flow tests [8,12–14], allowing the rapid detection of antibodies from small samples of blood. However, the serological tests, particularly the widely deployed CATT assay, have significant specificity and sensitivity issues [15,16], and neither can confirm the presence of trypanosomes. Such confirmation comes from subsequent microscopy of tissue fluids and is the current WHO recommended diagnostic criteria for drug treatment. Whilst more sensitive molecular diagnostics are available, such as those using the polymerase chain reaction (PCR), these techniques require invasive sampling, expensive equipment, cold chain for reagents, and training to produce reliable results [17] making them difficult to implement in rural regions with limited resources.

*Gambiense*-HAT case finding is therefore limited by the reach of screening activities and sensitivity of the current diagnostic tools, which may be missing infected individuals. One such emerging concern is those individuals who are persistently seropositive and can be positive by PCR, but who have no detectable blood parasitaemia and remain asymptomatic for a long period of time [18–21], as long as 29 years in one case [22]. These suspected latent infections, known as SERO TL+, is concordant with laboratory animal model experiments demonstrating that trypanosomes can be undetectable in the blood but sequester to other tissues of the mammalian host such as the skin, where they are made accessible to the tsetse fly during feeding [23]. It has been hypothesised that these skin-dwelling trypanosomes may represent a significant reservoir for transmission in humans, and indeed, in a recent field survey in Guinea, trypanosomes were detected in skin biopsies of both confirmed HAT cases and SERO TL+ individuals with no detectable blood parasitaemia [24]. Such latent infections could be a particular threat for the elimination goal set by WHO [21,25], as they fall below the current diagnostic criteria for treatment. Consequently, point of care, field appropriate diagnostics are essential to enable detection of all infected individuals, including latent infections, to ensure early treatment and facilitate disease elimination.

Such diagnostic tools could include the use of Raman spectroscopy. Raman spectroscopy is a portable [26–28], label-free and a non-invasive analytical technique that utilises the scattering of monochromatic light by a sample to produce a measurement of the chemically-specific vibrational fingerprint of the tissue [29]. This fingerprint can be altered by biological processes, such as an infection, which result in subtle changes to the chemical composition of the tissue. Thus, it might be predicted that an altered biological composition caused by the presence of trypanosome parasites in the skin could be detected using this technique. Raman has been used extensively for various types of biological studies including diagnostics, primarily focused on non-infectious diseases, such as cancer [26,30,31]. Recently, there has been an increasing awareness of the utility of this approach for the diagnosis of a range of infectious pathogens, e.g. sepsis, meningitis, hepatitis B or malaria [32–37], by examining *ex vivo* samples. However, it has not been used for the detection of pathogens *in vivo* or been applied to the diagnosis of African Trypanosomiasis.

This study evaluates the capability of Raman spectroscopy in detection and diagnosis of African trypanosomiasis by probing the skin. In this study, we report the signature Raman spectrum of *Trypanosoma brucei* and demonstrate that it is possible to detect, *ex vivo* and *in situ*, skin samples from *T*. *b*. *brucei*-infected animals in a murine model of African trypanosomiasis. Raman spectroscopy using multivariate data analysis, demonstrates the significant potential of Raman as a tool for non-invasive diagnosis of parasitic infection from the skin and sets the groundwork for the detection of *T*. *b*. *gambiense* in human patients in the field.

## Results

### *Trypanosoma brucei* biological characterisation

**Fig 1:**
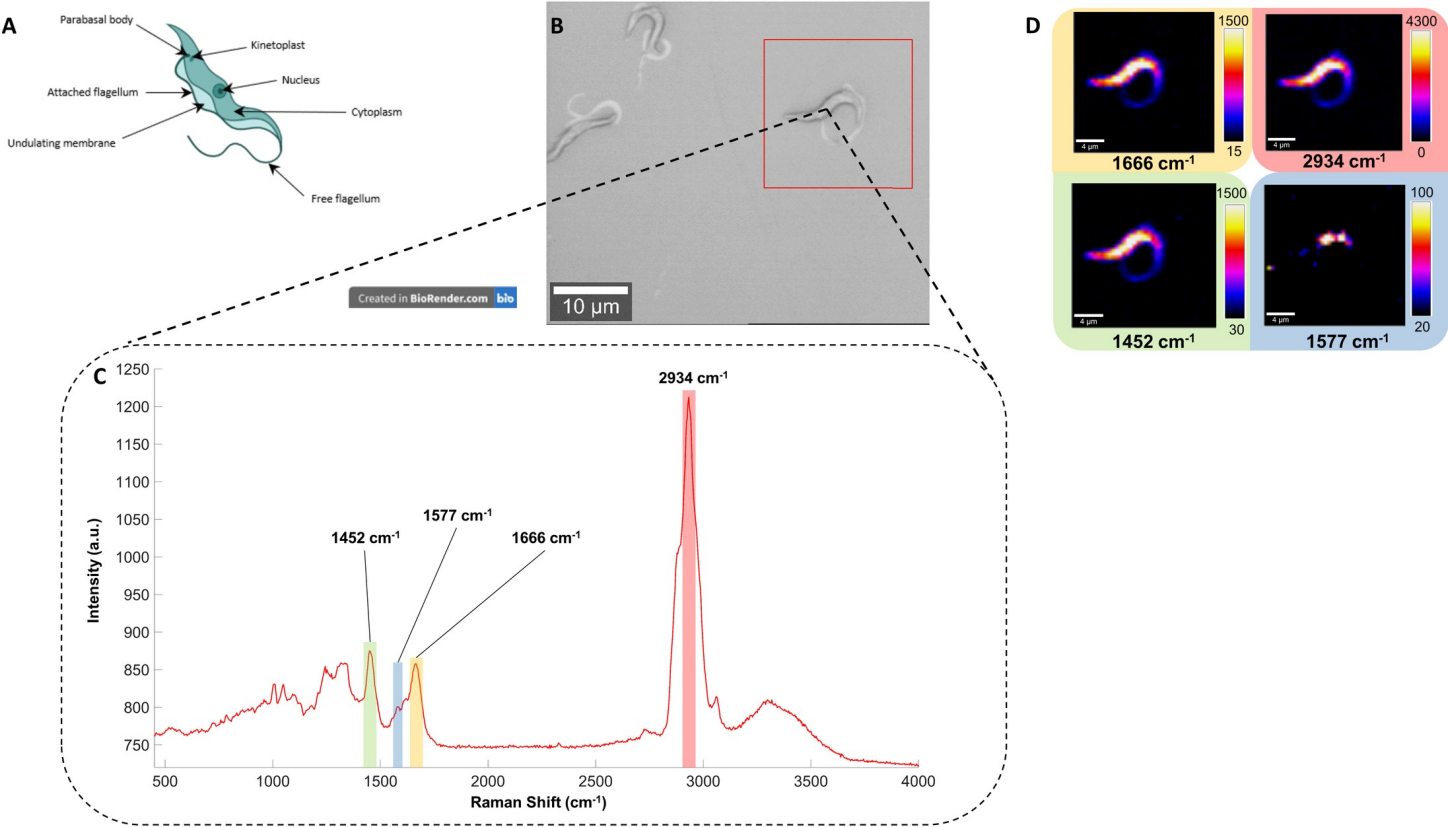
Raman spectrum of *Trypanosoma brucei brucei* strain STIB247 and some examples of its associated false colour image obtained with Raman measurements. Data obtained with a 532 nm laser, 6 s acquisition time and 100x lens. (A) A schematic of a bloodstream trypanosome (Created in BioRender.com), (B) white light image of *T. b. brucei* mapped where the red square correspond to 25 x 25 μm mapped at 0.5 μm step size, (C) representative Raman spectrum of the parasite, taken from a single point on the parasite, and (D) false coloured image associated of the Raman peaks 1452, 1577, 1666 and 2934 cm^-1^ with their respective intensity bar, which assign a colour gradient from the lowest (black) to the highest (white) intensity.

To determine the parasite’s molecular composition and generate a Raman fingerprint we examined cultured bloodstream form *T*. *b*. *brucei* parasites using a 532 nm laser excitation confocal Raman microscope. A schematic of the cell structure of a representative bloodstream form trypanosome is depicted in Fig 1A. We mapped the parasites by taking a single Raman measurement at 0.5 μm intervals across the trypanosome cell (Fig 1B and 1C). By using the Raman intensity of an identified peak, corresponding to an assigned biological vibration, a false colour Raman intensity map was obtained showing the distribution of the vibration throughout the parasite. Fig 1D shows examples of Raman peak intensity maps for four Raman peaks (1452, 1577, 1666 and 2934 cm^-1^). The resulting intensity maps for other Raman peaks are given in S1 Fig.

*Trypanosoma brucei* parasites are covered in a dense glycoprotein coat, composed of approximately 10^7^ identical copies of variable surface glycoprotein (VSG) which makes up 95% of the surface protein and can be frequently switched for a different VSG from its vast genomic repertoire, through a process of antigenic variation [38,39]. By mapping the trypanosome, it was possible to elucidate Raman vibrations associated with the VSG and other cellular components. To investigate the different biological components present in the parasite, a tentative assignment to match Raman peaks to specific molecular vibration associated with various biological components of each of the different Raman peaks was performed. Raman spectroscopy provides vibrationally specific information with the energy (Raman Shift) directly linked to a molecular group vibration [29]. However, in a complex matrix such as a biological sample it becomes increasingly difficult to assign Raman peaks to a specific group as there are often multiple contributions to the peak in the spectrum. Thus, previously published works are used to assign Raman peaks to specific biological components and this approach is commonly referred to as a tentative assignment. This approach offers a potential framework for the measurements obtained by Raman spectroscopy and helps develop hypotheses on the biochemical changes observed in the skin due to an infection. The tentative Raman assignments for the analysis of *Trypanosoma brucei* are summarised in Table 1.

**Table 1:** Tentative assignment of Raman peaks for the characterisation of the biological composition of *T. b. brucei* using previously published works from biological samples [31,41,42].

Raman Shift (cm^-1^)	Tentative assignments of Raman peaks	Raman Shift (cm^-1^)	Tentative assignments of Raman peaks
**3305**	O-H stretch	**1095**	C-C stretch or phosphodioxy group (PO_2_^-^)
**3065**	C-H stretch	**1048**	C-C stretch proline or PO_4_^3-^ symmetric stretch
**2934**	CH_2_ asymmetric stretch	**1005**	Phenylalanine
**2876**	CH_2_ symmetric stretch	**953**	CH_3_ of α-helical proteins
**1666**	Amide I	**860**	Phosphate group or tyrosine
**1577**	Nucleic acid modes	**780**	Phosphate group (DNA/RNA)
**1452**	CH_2_CH_3_ deformation	**725**	C-S protein
**1325**	CH_2_CH_3_ wagging mode in proteins and DNA	**658**	C-S stretch of cystine
**1244**	Amide III	**525**	Phosphatidylserine or S-S disulfide stretch

The largest peak in the spectrum at 2934 cm^-1^ (denoted in red in Fig 1C) was assigned to a C-H stretch of lipids/proteins. The Raman peaks observed at 1666 cm^-1^ (highlighted in yellow in Fig 1C) and 1244 cm^-1^, were assigned to Amide III and Amide I vibrations, respectively. Two small Raman peaks could also be observed at the base of the Raman peak 1666 cm^-1^ for the Amide I and were located at 1583 and 1619 cm^-1^. They were tentatively assigned to phenylalanine and tyrosine residues within the parasite [40]. These molecular groups, as well as the presence of another peak associated with CH_2_CH_3_ deformation at 1452 cm^-1^ (highlighted in green in Fig 1C), are ubiquitous in proteins and are consistent with long chain amino-acid formed by the VSG around the surface of the trypanosomes.

The Raman peak at 1577 cm^-1^ (denoted in blue in Fig 1C) related to a nucleic acid vibration indicates the detection of DNA or RNA residues within the parasites. This signal was not dispersed across the parasite but localised to a small area on the Raman intensity map (Fig 1D), indicative of the confinement within the nucleus. Further, evidence supporting the detection of the nucleus is also indicated by the Raman peak at 780 cm^-1^ (S1 Fig), which is characteristic of the phosphate backbone of DNA and produces a similar distribution on the intensity map. Other Raman peaks associated with phosphate groups positioned at 1048, 1095 or 1325 cm^-1^ (S1 Fig) indicate a large distribution of this molecular group across the parasite and suggests that their probable source is phospholipids.

The VSG coat protein is attached to the membrane of the parasite via a glycosylphosphatidylinositol (GPI) anchor [43], therefore we expect it to be present in large quantities, detectable using Raman spectroscopy. The Raman peaks at 1048 cm^-1^ (PO_4_^3-^) and 1095 cm^-1^ (PO_2_^-^) (tentatively assigned as phospholipids) were observed across the entire surface of the trypanosomes (S1 Fig), suggesting that the GPI and/or phospholipids can be identified. Additionally, the peak at 525 cm^-1^, tentatively assigned to phosphatidylserine was observed through the surface of the main body of the parasite, suggesting that phospholipids contained in the cell membrane could also be tracked on the surface of the parasite, despite the dense VSG layer. Indeed, the *T*. *b*. *brucei* surface composition contains numerous fatty acids, lipid and phospholipid residues on its membrane such as phosphatidylcholine, phosphatidylethanolamine, phosphatidylinositol or phosphatidylserine [44,45]. Thus, the Raman peaks assigned to phosphate groups could therefore be associated with the phospholipid layer present in the membrane of the trypanosomes as well as the GPI anchor.

The biological information obtained via the characterisation of the different Raman peaks provides a vital understanding of the molecular composition of *T*. *b*. *brucei*. Suspected Raman peaks related to the abundant VSG protein coat (e.g. 1666 and 2934 cm^-1^), GPI anchor or phospholipids layer (e.g. 1048 cm^-1^) covering the parasite were identified as well as those marking organelles such as the nucleus (780 and 1577 cm^-1^). This analysis also provided a unique Raman fingerprint for *T*. *b*. *brucei*, which could be used as a reference for the investigation of infected murine skin.

### Raman spectra of *T*. *b*. *brucei* -infected versus uninfected *ex vivo* skin tissue

To examine the utility of Raman spectroscopy to detect skin-dwelling trypanosomes we used archived samples from a previously published study of skin dynamics in a BALB/c murine model of *T*. *brucei* infection [23]. A skin sample was selected from a time point of infection (day 15) in which parasites were detectable in the blood (~6x10^7^ parasites/mL) and confirmed by two independent histologists to also be present at moderate levels in the extravascular tissues of the skin [23]. Due to the potential interference of the stained antibodies with the Raman signal, stained skin sections were not used with the Raman instrumentation but measurements were instead performed on new histological sections prepared from the sample previously shown to possess extravascular parasites [23]. The skin sections were fixed in formalin and mounted on Raman grade–calcium fluoride (CaF_2_) slides. The skin sections were mapped with a confocal Raman microscope, and seven maps were acquired across each section from both *T*. *b*. *brucei*-infected and uninfected skin. The average of the Raman maps for each skin section were compared using principal component analysis to assess the spectral differences between infected and uninfected skin.

**Fig 2:**
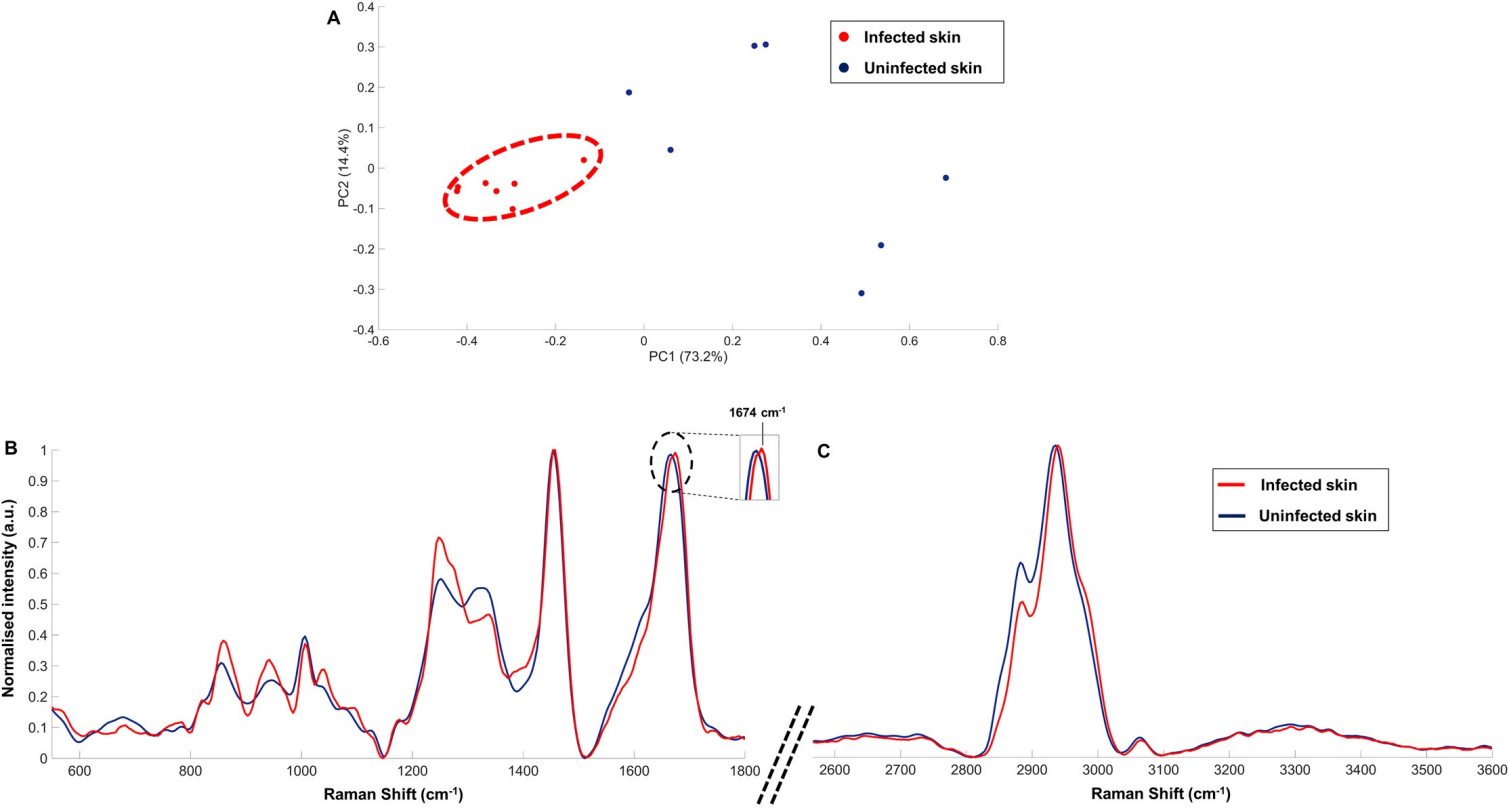
Raman analysis of *T*. *b*. *brucei* infected (red) and uninfected (blue) murine skin sections (BALB/C) on CaF_2_ slides showing (A) the resulting PCA scores plot, and their associated averaged normalised Raman spectra in the spectral window (B) 550–1800 cm^-1^ and (C) 2550–3600 cm^-1^. Raman maps measurements were taken using a 532 nm excitation laser and 1 s acquisition time over a 50 x 50 μm square on the skin for each map and 2 μm step across x and y. Spectra were obtained by averaging seven maps from each tissue sample, then normalised to the highest peak intensity. Tentative peak assignment is given in Table 2.

The resulting principal component analysis (PCA) plot (Fig 2A) displays a clear separation across PC1 (73.2%) between the maps derived from infected skin sections (red dots) compared to those derived from uninfected skin sections (blue dots). In addition, the seven maps derived from *T*. *b*. *brucei* infected skin form a distinct cluster, that differentiate them from non-infected tissue. This suggests that infection of the skin with trypanosomes caused specific and homogeneous alterations to the molecular composition of the tissue, despite a level of heterogeneity in the uninfected tissue. As the skin is a complex matrix, this homogeneity was interesting, indicating a specific molecular change across the tissue that could be detected with Raman. PC1 and PC2 spectra, describing the spectral differences between infected and uninfected skin sections, are shown in S2 Fig. The loading spectrum for PC1 revealed that the main Raman peaks that explains the spectral differentiation were characteristic of the C-H vibration (2953 and 2989 cm^-1^) for lipids and proteins. Other Raman peaks such as 942 (C-C backbone or proline), 1248 (Amide III) and 1392 (C-N stretching) cm^-1^, suggests an alteration in the protein contents of the skin matrix.

To understand and interpret the biological variations during an infection, each data set were averaged into a single spectrum and displayed in Fig 2B and 2C. These two fingerprints provide a more detailed overview of the biological variation between infected and non-infected skin across a larger section of the sample and the tentative assignment of specific Raman peaks allowed the identification of the possible biological changes induced by the presence of the parasites. The direct spectral comparison of the infected and uninfected Raman spectra (Fig 2B and 2C) supported the PCA clustering (Fig 2A); with *T*. *b*. *brucei*-infected skin spectrally differentiated from the uninfected state. The standard deviation of these two averaged spectra for infected and uninfected skin were calculated before the spectra were normalised and showed in S3 Fig. It was observed that the variation of Raman signal for the infected skin was smaller than for uninfected skin, in good agreement with the distinct cluster observed for the infected skin data points in the PCA scores plot. Taken together, these results suggest that the biological changes induced by the *T*. *b*. *brucei* infection are homogenously spread across the skin.

A tentative assignment of the Raman peaks specific to the infection was performed and grouped in Table 2. No unique peak specific to the infection was detected. Instead, a variation in intensity was observed between the two sample types, across the spectral range, suggesting a parasite-induced disruption to the skin composition, that may result from the presence of parasites in the skin, parasite-derived extracellular factors, local immune responses, or most likely, a combination of these variables.

**Table 2:** Tentative assignment of Raman peaks specific to the trypanosome infected skin, based on Fig 2B and 2C. Specific Raman peak were chosen when they showed a clear shift (s), decrease in intensity (d) or increase of intensity (i) compared to the uninfected skin Raman spectrum. The tentative biological assignment was performed using previously published works from biological tissues. [31,40–42,46].

Raman peaks of infected skin spectrum compared to uninfected (cm^-1^)	Tentative assignment of Raman peaks
**3062** ^**d**^	Lipids
**2972–3000** ^**i,s**^	Cholesterol, cholesterol ester, lipids, fatty acid
**2939** ^**s**^	C-H stretch lipids/proteins
**2885** ^**d**^	CH_3_, lipids or fatty acid
**1674** ^**i**^	Amide I or cholesterol
**1580–1630** ^**d**^	Phenylalanine and tyrosine
**1339** ^**d**^	CH_2_CH_3_ wagging mode
**1273** ^**i**^	Amide III
**1248** ^**i**^	Amide III
**1096** ^**i**^	PO_2_^-^ stretch
**1041** ^**i**^	C-C stretch proline or PO_4_^3-^ stretch
**1006** ^**d**^	Phenylalanine
**942** ^**i**^	C-C backbone or proline
**859** ^**i**^	Phosphate group or tyrosine
**820** ^**i**^	PO_2_^-^ stretch (DNA)
**785** ^**i**^	Backbone O-P-O

The main spectral differences between the infected and uninfected skin section were associated with lipids and proteins. Raman peaks variation was observed in the C-H region of the spectra between 2800 and 3062 cm^-1^. This alteration of protein/lipid composition was also supported by the contribution of other Raman bands at 942 cm^-1^ (C-C stretch backbone, proline), 1248 cm^-1^ (Amide III), 1273 cm^-1^ (Amide III α-helix) or 1339 cm^-1^ (CH_2_CH_3_ wagging mode). These Raman peaks all displayed an increased in intensity, or a shift for 2939 cm^-1^ and the region 2972–3000 cm^-1^ for example, in the spectrum related to the infection. Other Raman peaks specific to the infection at 785 cm^-1^ (backbone O-P-O), 820 cm^-1^ (PO_2-_ stretch), 859 cm^-1^ (phosphate group or tyrosine), 1041 cm^-1^ (C-C stretch proline, PO_4_^3-^ stretch) or 1096 cm^-1^ (PO_2-_ stretch) indicated a variation in the phospholipids or DNA composition of the skin.

To assess the specificity of the Raman skin spectra to indicate trypanosome infections, we compared skin from mice infected with either *T*. *b*. *brucei* or *Plasmodium berghei*, a rodent model of malaria that has been detected in subcutaneous sections of the skin [47]. Skin from a *P*. *berghei* infected mouse was analysed using the same method as for *T*. *brucei*, alongside uninfected skin from an appropriately matched control mouse. For skin samples from both *T*. *brucei* and *P*. *berghei* infected mice, Raman spectra for the respective control samples were subtracted to enrich for Raman signal specific to the infection, and the resulting spectra compared (S4 Fig). The comparison revealed contrasting intensities in a number of Raman peaks indicating that skin from a trypanosome infected mouse could be differentiated from that from a *P*. *berghei* infected mouse by Raman spectroscopy. The key differences between the infections observed in the spectra, shown in S4 Fig, were related to the lipids/protein composition with a notable shift in the C-H Raman peak for the trypanosome infected mouse whereas no shift was observed in the *P*. *berghei* infected mouse. Moreover, four intense Raman peaks (S4 Fig) were distinctly different between infections. Taken together, the biological changes induced in the skin by infection with *T*. *brucei* or *P*. *berghei* result in a different Raman signal measured.

The results shown in Fig 2 confirmed a clear differentiation between the skin from trypanosome infected and uninfected control mice, with a homogeneous spectral fingerprint defining trypanosome infection. The tentative assignment of the Raman peaks specific to the *T*. *brucei* infection and characterisation of the biological composition of the *T*. *brucei* infected skin, suggest a protein/lipid rearrangement within the skin matrix either directly or indirectly induced by the presence of trypanosomes which is detectable by Raman spectroscopy. Additionally, the Raman signal in mouse skin for the trypanosome infection was found to be distinct from a *P*. *berghei* infection, supporting the possibility of detecting pathogen-specific biological changes to the skin by Raman spectroscopy.

### Comparison between Raman spectra of purified bloodstream *T*. *b*. *brucei* and infected and uninfected *ex vivo* murine skin sections

The characterisation and differentiation of *T*. *brucei* infected skin sections compared to uninfected skin sections *ex vivo* (Fig 2) established the possibility of a detection method targeting the skin. Next, it was important to determine whether the presence of the parasite was directly detected by Raman and strongly involved in the spectral differentiation or if the biological changes of the skin matrix induced by its presence were the main factor of discrimination.

**Fig 3:**
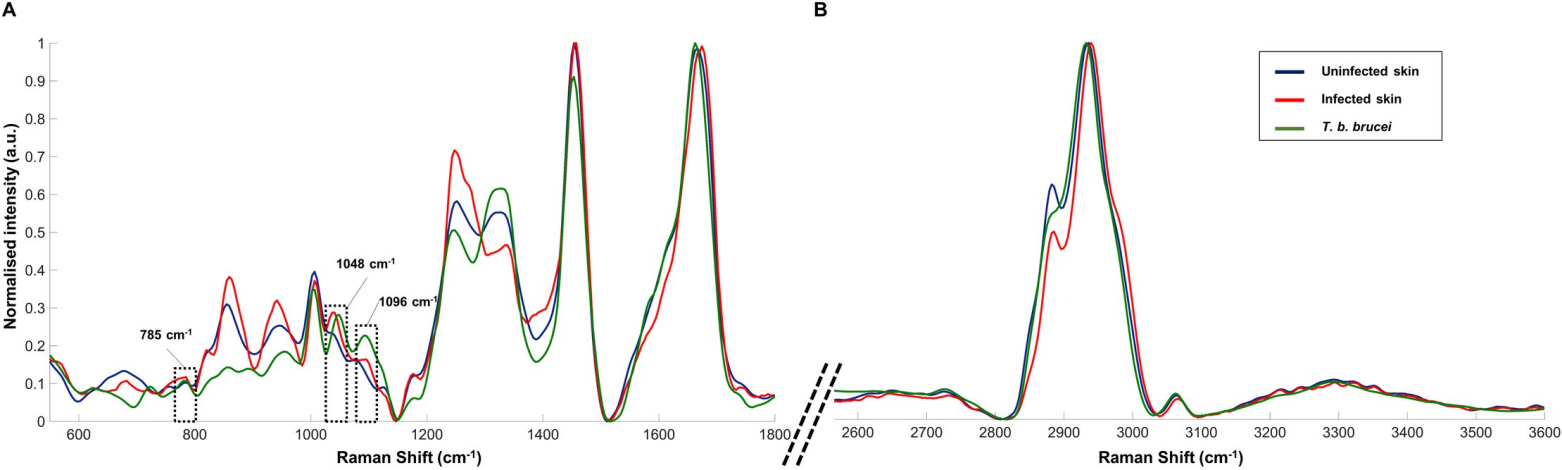
Spectral comparison between reference spectrum of *T*. *b*. *brucei* and uninfected and *T*. *b*. *brucei* murine infected skin (BALB/C). Five different *T. b. brucei* were mapped and averaged into a single Raman spectrum and both skin spectra were shown in Fig 2. Two Raman spectral region are shown: (A) 550–1800 cm^-1^ and (B) 2550–3600 cm^-1^.

Fig 3 shows the comparison between the Raman fingerprint for both region 550–1800 cm^-1^ (A) and 2550–3600 cm^-1^ (B) of cultured *T*. *b*. *brucei* parasites (five maps from different *T*. *b*. *brucei* were averaged into a single Raman spectrum) with infected and uninfected skin samples obtained from the *ex vivo* experiment (Fig 2B and 2C). The resulting standard deviation for the *T*. *b*. *brucei* Raman spectrum is shown in S5 Fig. This qualitative comparison between each spectrum allows us to investigate the contribution of the parasite’s components in the discrimination of the infected skin.

Several Raman peaks displayed increased intensity in both the fingerprint of *T*. *b*. *brucei* and infected skin relative to uninfected tissue. Raman peaks at 1041 and 1096 cm^-1^ for the infected skin closely matched the two peaks observed in the Raman fingerprint of the purified parasite (1048 and 1095 cm^-1^). They both displayed an increase in intensity compared to the uninfected skin, which suggest a direct contribution of the parasite to the infection-specific Raman spectra. They were previously assigned to phosphate group vibrations (Tables 1 and 2). The presence of parasites in the skin (in particular the phospholipids on their surface) results in an increased signal intensity from these peaks, thus, these peaks could represent the presence of parasite in the skin or an increase in secreted parasites’ protein concentration in the skin such as soluble VSGs. Additionally, the Raman peak at 785 cm^-1^ (PO_2_^-^ backbone for DNA) displays an increase in intensity within the infected skin spectrum (denoted in red in Fig 3) and align with the peak observed on the purified trypanosome spectrum (denoted in green in Fig 3) supporting an increase in cell density within the infected tissues, relative to controls.

However, most Raman peaks that differed in intensity between infected and uninfected skin did not correspond to peaks detected in purified parasites. Variation in the VSGs composition of the surface coat between sampled parasite populations due to VSG switching, a strategy of immune evasion characteristic of *T*. *brucei*, unlikely explain these spectral peak differences. This is because the subtle changes between expressed VSG variants, while immunogenically distinct, would not be detectable by Raman spectroscopy in such a complex matrix. Additionally, the three Raman peaks identified here at 785 (PO_2_^-^ backbone for DNA), 1041 and 1096 (phosphate group) cm^-1^, although aligned with the spectral fingerprint of the trypanosome, may have alternative sources in the host due to changes to proteins or cell types in the skin matrix induced by the infection. Therefore, the majority of discriminating Raman peaks between *T*. *brucei* infected and uninfected skin cannot be attributed directly to the detection of the parasite but related to the measurement of a general change in the composition of the skin due to the presence of parasites in the host.

### Raman analysis of *T*. *b*. *brucei* infected versus uninfected skin *in situ*

We have demonstrated that skin sections taken from a *T*. *brucei*-infected mouse could be spectrally differentiated *ex vivo* using Raman spectroscopy. To take this technique further with a view to performing analysis *in vivo* on live subjects in the field, we carried out analysis using a portable Raman instrument equipped with a lower energy excitation wavelength of 785 nm (S6 Fig), appropriate for *in vivo* field use. The following experiments were performed *in situ* using euthanized mice.

**Fig 4:**
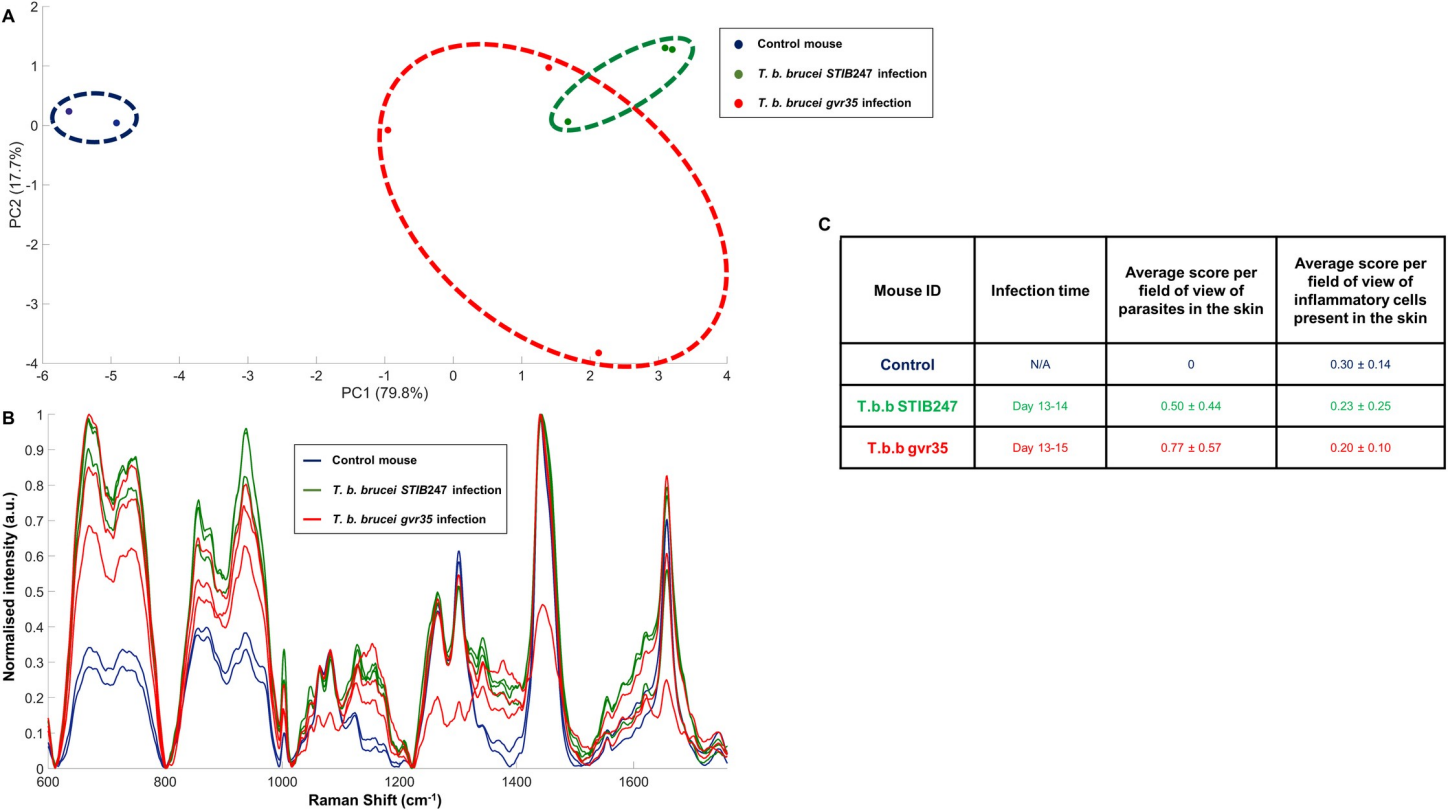
*In situ* Raman analysis of the skin of uninfected and *T*. *brucei* infected mice (BALB/C) at 14 days post-infection. Measurements were obtained using a 785 nm excitation laser with a 60 s acquisition time and ten measurements were taken across each murine skin. The resulting principal component analysis scores plot is shown in (A) where each dot corresponds to the averaged (from the 10 Raman measurements per sample) and normalised spectrum of one murine skin sample, (B) displays the spectral comparison of each Raman spectrum and (C) table regrouping the mean data and its SD from the semi-quantitative measurements of parasites and inflammatory cells in the skin stained by immunochemistry; all data from the immunochemistry staining are grouped in S9 and S10 Figs.

To test for variation in Raman spectra within and between strains of *T*. *b*. *brucei*, groups of three mice were infected with two commonly used laboratory strains: STIB247 or GVR35. The mice were euthanised between day 13–15 post infection, when parasites are detectable in both the blood and the skin and compared with two matched uninfected controls. Mice infected with STIB247 strain were euthanised at day 13 (two mice) and day 14 (one mouse), and mice infected with GVR35 strain were euthanised at day 13 (two mice) and day 15 (one mouse). This delay between the time points was necessary to account for the time needed to acquire Raman data in one day and to only analyse fresh skin samples. Ten Raman measurements were acquired from the abdominal region of the mice using the portable Raman instrument. The resulting spectra were averaged, normalised and presented in Fig 4 together with a PCA score plot which demonstrates clear differentiation between uninfected and infected mice (Fig 4A and 4B). Each coloured marker on the scores plot corresponds to the ten averaged Raman measurements. Both infections were well separated from the controls across PC1 (79.8%) indicating that *T*. *b*. *brucei* infection can be clearly measured and differentiated from the control group. This also indicates a level of similarity in the chemical fingerprint of *T*. *b*. *brucei* infection both *between* and *within* parasite strains compared to uninfected mice. The loadings spectra associated with PC1 and PC2 are shown in S7 Fig. The Raman spectra produced by the 785 nm excitation wavelength instrument are not directly comparable with the 532 nm excitation wavelength due to variance in Raman peak intensity produced by the different excitation wavelength as well as the different resolution between the two instruments. These results demonstrate that using a higher excitation wavelength contained within a portable form factor still permits the distinction of infected from uninfected tissues.

To gain insight into the potential biological mechanisms underlying the observed differences between infected and uninfected mice, a semi-quantitative measurement of the parasite burden in the skin as well as inflammatory cells was performed by immunostaining. An example of trypanosome immunostaining of infected and uninfected skin samples for the assessment of parasite burden is shown in S8 Fig. The scores were recorded across ten random fields of the skin section at 40x (parasite burden) or 20x (inflammation) magnification and the mean and their associated standard deviation (SD) displayed in Fig 4C, with the raw data in S9 and S10 Figs. Both intra- and extra-vascular parasites were evident in the skin of the two *T*. *b*. *brucei* infection models by day 14, with a similar mild-to-moderate parasite burden (≤50 parasites/field). This parasite skin burden for *T*. *b*. *brucei* STIB247 is consistent with that reported by Capewell *et al*., for STIB247 infection in BALB/C mice at day 12 and 15 [23]. However, as Capewell *et al*., reported in the same study, infection of the skin with *T*. *b*. *brucei* was not associated with a major inflammatory response (Fig 4C). Neither *T*. *b*. *brucei* strains had an average inflammatory cell score higher than the uninfected mice, by semi quantitative scoring. The lack of skin inflammation observed in the infected skin sections both between and within the strains, adds support to the hypothesis that differentiation of the Raman spectra between the infected and uninfected tissues is related to non-immune mediated changes to the biological composition of infected skin.

### Raman spectroscopy time course of the skin in *T*. *b*. *brucei* mouse model

A time course experiment was performed to determine how the Raman spectra of the skin changes throughout the course of infection in the *T*. *b*. *brucei* mouse model and to obtain preliminary data on how early in infection detectable changes to the skin occur. This experiment was performed as for the previous *in situ* study, using *T*. *b*. *brucei* STIB247, but substituting the albino BALB/c mouse strain for the dark-haired C57/Black6J mouse strain, to determine the effect of melanin on Raman targeting of the skin for detection of trypanosome infection. The spectra obtained were similar from both uninfected BALB/C and C57/black6J control mice (S11 Fig). indicating that melanin in the skin did not markedly affect the Raman measurements.

**Fig 5:**
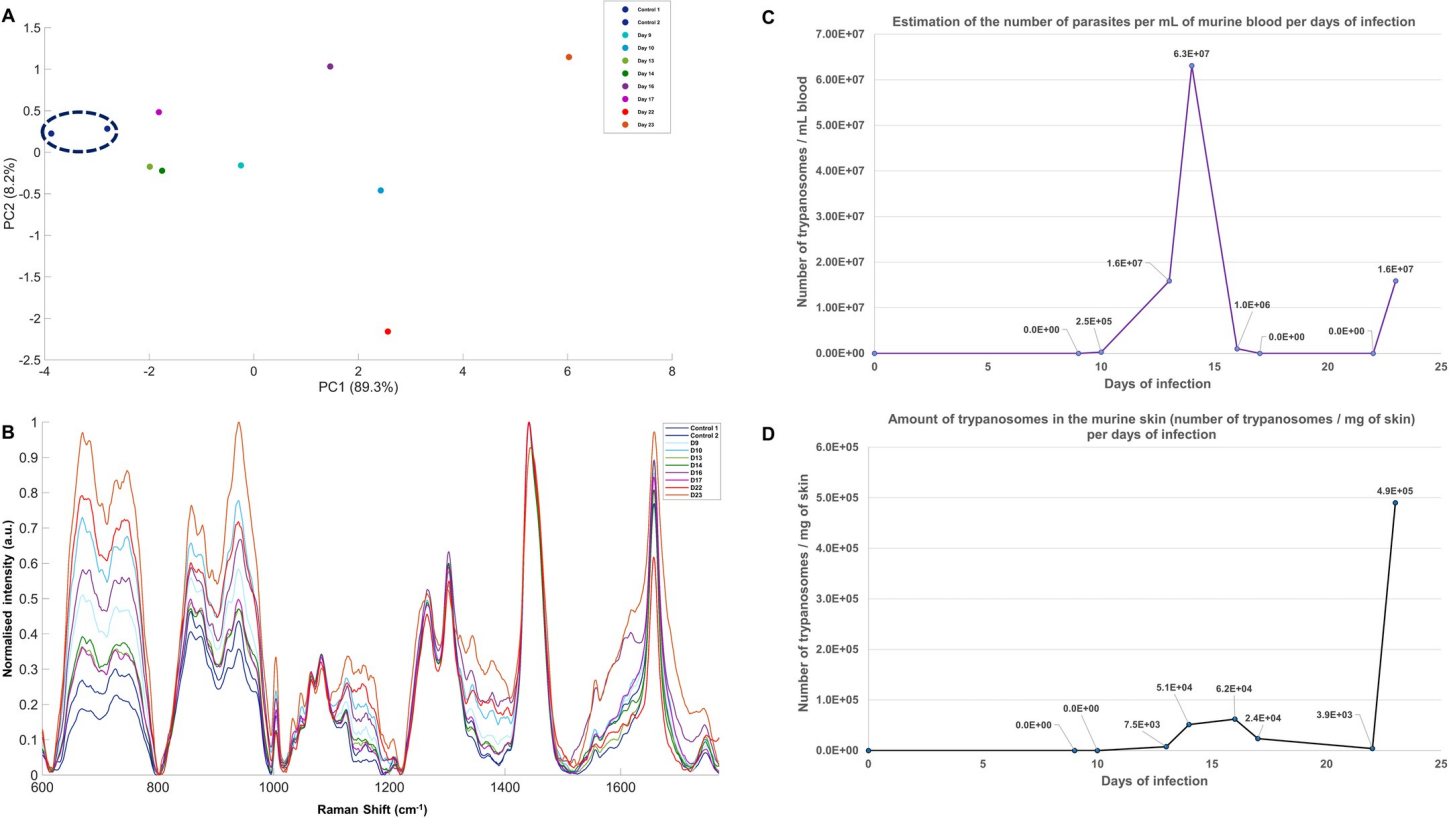
*In Situ* Raman analysis of a *T*. *b*. *brucei* infection time course in C57/black6J mice compared to detectable blood parasitaemia and skin parasite burden. (A) PCA scores plot, (B) spectral comparison of each averaged and normalised Raman spectrum, (C) estimation of the blood parasitaemia and (D) estimated number of parasites/ mg skin by qPCR for PFR2 gene over the same time course as the Raman measurements. Raman measurements were obtained using a 785 nm excitation laser with a 30 s acquisition time and five measurements were taken across each skin sample.

For infected mice, parasitaemia measurements were taken daily, and mice were euthanised and examined by Raman spectroscopy at different time points after the infection (day 9, 10, 13, 14, 16, 17, 22 and 23). Raman spectra were obtained from the abdominal skin at each timepoint. Five measurements were made across the skin area and averaged into a single Raman spectrum and normalised prior to analysis by PCA (Fig 5A). The PCA scores plot showed clear differentiation between infected time point at day 10, 16, 22 and 23 and uninfected skin across PC1 (89.3%). Their associated loading plots are shown in S12 Fig. Both late time points day 22 and 23 were well discriminated from the control group across the PCA score plot, suggesting that the biochemical composition of the skin is strongly affected by the infection at a later stage. Nonetheless, the discrimination of the time point day 10 and 16, and to a lesser extent day 9 from uninfected, suggests that infection related changes to the skin were also detected by Raman earlier in infection. The averaged and normalised Raman spectrum for each time point is plotted in Fig 5B.

Parasitaemia over the time course, with its characteristic peaks and troughs of *T*. *brucei* infection are shown in Fig 5C, with the caveat that estimation of parasitaemia by microscopy had a limit of detection of ~10^5^ trypanosomes/mL. To quantify parasite load in the skin we used a quantitative PCR (qPCR) approach to detect copies of *T*. *brucei* paraflagellar rod protein 2 (PFR2) gene [48]. This approach, unlike the semi-quantitative scoring of histological slides performed in Figs 4 and S9 and S10, does not distinguish the presence of trypanosomes in the extravascular and intravascular space, therefore DNA was extracted from perfused skin to allow the accurate quantification of extravascular trypanosomes only.

Typically, parasites in the skin appear after an infection has been established in the blood, and have also been reported to undergo cyclic fluctuations, though to a lesser extent than parasitaemia [23]. Here, we first detected parasite in the skin by qPCR at day 13 (Fig 5D), three days after parasites were first detected in the blood at day 10 (Fig 5C). It is however possible that trypanosomes entered the skin earlier than day 13, as only a small section of skin was analysed and parasites might have been present in a different region of the skin. Their presence in the skin appeared to show fluctuations, that roughly correlate with those observed in the blood, with a strong second peak emerging by the final time point (day 23) in both tissues. This correlate well with the observation made with the PCA scores plot (Fig 5A) where data from day 23 were strongly discriminated from the control data points. An increased number of parasites in the skin was associated with a strong change in the biochemical composition of the skin, which was detected by Raman spectroscopy.

This differentiation was strongest at the latest stage of infection (day 23) when parasite density in the skin was at its highest. However, infected mice could be discriminated from the uninfected control mice from as early as day 10, three days before parasites were detectable by qPCR, suggesting biological changes to the skin are evident early in infection, and precede detectable parasite levels in the skin but match the first detectable parasites in the blood. Between the earlier and latest time points, the PCA indicates a non-linear relationship in Raman spectral changes, perhaps reflects fluctuations in parasite density observed in both parasitaemia and skin parasite burden and the biological changes induced by their presence (Fig 5C and 5D). This fluctuation can also be observed in the PCA scores plot where time points day 13, 14 and 17 were not differentiated from the control group.

**Fig 6:**
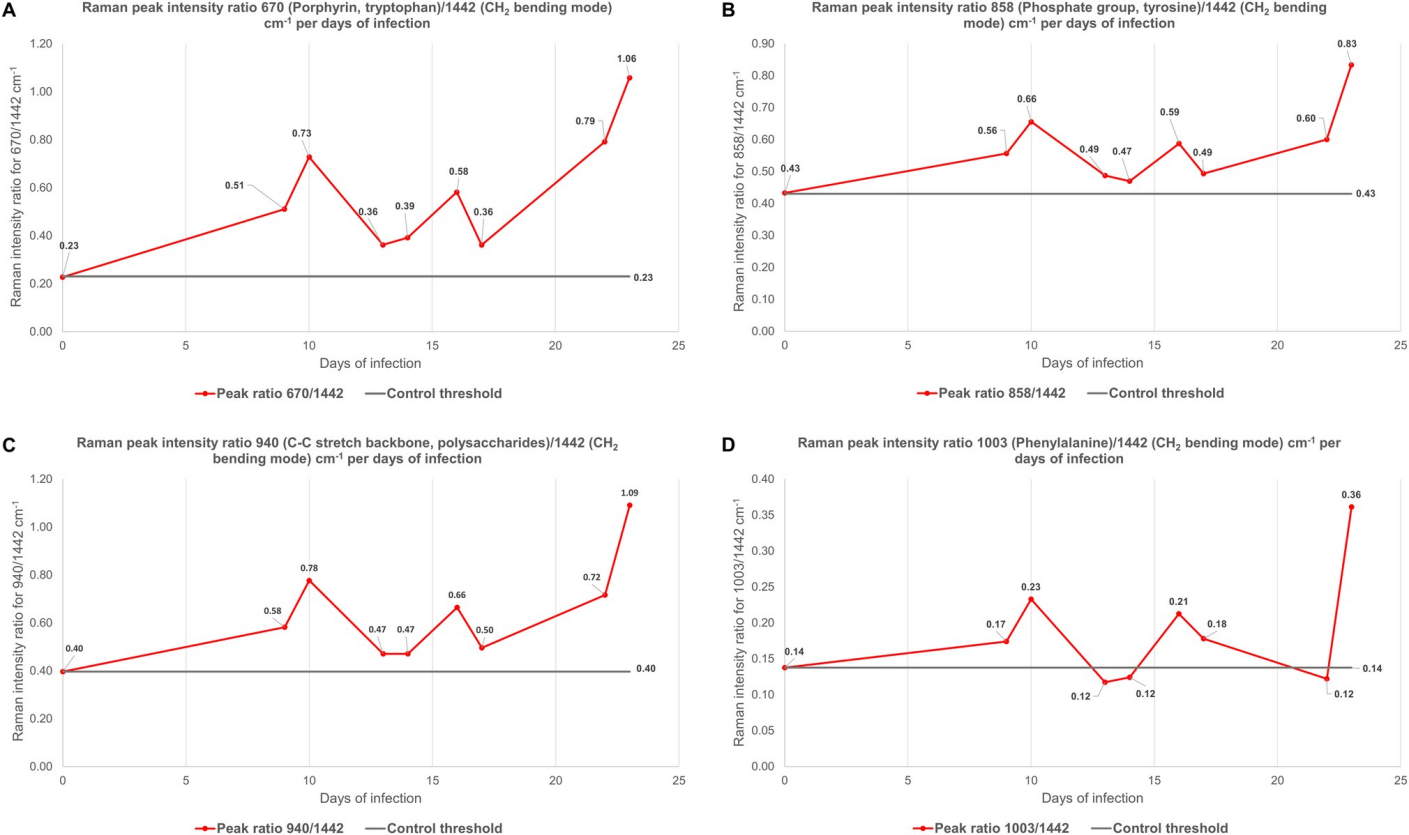
Graphs showing the evolution of the *T*. *b*. *brucei* infection in C57/black6J mice over the time course using a Raman peak intensity ratio. Four Raman peaks are displayed: (A) 670 cm^-1^ (Porphyrin, tryptophan) / 1442 cm^-1^ (CH_2_ bending mode), (B) 858 cm^-1^ (Phosphate group, tyrosine) / 1442 cm^-1^ (CH_2_ bending mode), (C) 940 cm^-1^ (C-C stretch backbone, polysaccharides) / 1442 cm^-1^ (CH_2_ bending mode) and (D) 1003 cm^-1^ (Phenylalanine) / 1442 cm^-1^ (CH_2_ bending mode). The grey line corresponds to the Raman peak ratio averaged between both control murine and act as a threshold for the detection of the infection.

To investigate the significance of individual spectral peaks in the detection of the infection at each time point a Raman peak ratio was determined by comparing a non-varying Raman peak (1442 cm^-1^(CH_2_ bending)) with each of the discriminating peaks in the spectrum. Examples of the Raman peaks ratio over the time course for four of the main Raman peaks are shown in Fig 6. The average of the Raman peak ratio for the skin from uninfected control mice is represented by a grey line and indicate a threshold for the detection of disease. Raman peak intensity ratios found above this threshold, were considered indicative of a *T*. *b*. *brucei* infection.

The four Raman peaks intensity ratios (670, 858, 940 and 1003 cm^-1^), displayed in Fig 6, showed an increase above the control threshold at the earliest timepoint of day 9, suggesting that despite an absence of detectable parasites in the skin by qPCR (Fig 5D), there were biological features associated with *T*. *b*. *brucei* infection that could be detected by Raman spectroscopy. However, it should be noted that when taking Raman measurements from the skin, some signal may also originate from parasites and cells in blood capillaries which would not be present in the perfused skin samples used for qPCR. Furthermore, the intensity ratio, for 670, 858 and 940 cm^-1^, did not fall back down to below the control threshold for the rest of the time course but did display a cyclic pattern that reflect the observed fluctuations of parasite numbers in the blood and skin. Therefore, their respective associated vibrations appear to be indicative of the number of trypanosomes in the host, either directly as a consequence of parasite numbers or through their interaction with the skin matrix. Ratios for other Raman peaks also appeared to show the same pattern over time, although the intensity ratio sometimes dipped below the control threshold (S13 Fig). Indeed, the Raman peak intensity ratio at 1003 cm^-1^ (phenylalanine), showed in Fig 6, and for example 1047 (proline, PO_4_^3-^ stretch) or 1248 cm^-1^ (Amide III) (S13 Fig) had their intensity ratio fall within the threshold at some point during the infection, despite detectable parasites levels in both the blood and the skin at those time points (day 13 and 14). This suggests that other biochemical processes of the parasite/host interaction are also measured by Raman over the course of infection. Indeed, despite finding some Raman peaks that appeared to follow the trend of parasite numbers in the skin measured by qPCR, it is important to look at the entirety of the Raman spectrum from the infected skin. As such, it was observed the infected skin Raman profile reflect a general biological change within the infected skin, which is caused by the presence of parasites but also by other biological mechanisms due to the infection. Ultimately, detecting these biochemical changes before the parasites enters the skin could be very valuable in the early diagnosis of sleeping sickness.

## Discussion

Here we have demonstrated that Raman spectroscopy of the skin can consistently differentiate infected from uninfected tissue in a *T*. *b*. *brucei* mouse model of African trypanosomiasis, under both *ex vivo* and *in situ* conditions. While for *ex vivo*, a confocal Raman microscope using 532 nm excitation provided a more sensitive approach to detect the differences induced in the skin by trypanosome infection, the change to a less sensitive Raman system with a lower energy laser (785 nm excitation wavelength) and no confocal setting, but more portable instrument, for *in situ* experiments established that this differentiation remained robust, thus opening up options for potential use in the field.

The spectral changes induced by *T*. *b*. *brucei* infection appeared consistent between experimental approaches and coupled with tentative assignment of key Raman peaks, can offer insight into changes to the chemical composition of the skin associated with African trypanosomiasis. The spectral window between 800 and 1000 cm^-1^ showed consistent changes of peak intensities between *ex vivo* (Fig 2) and *in situ* (Fig 4) studies. The Raman peaks around 858 (phosphate group, tyrosine), 878 (hydroxyproline, tryptophan) and 940 (C-C stretch backbone, proline) cm^-1^ all displayed an increase in intensity with *T*. *brucei* infection. The tentative assignment of these peaks demonstrates the enrichment of phosphate groups and amino acids, which originates from an increase or rearrangement of phospholipids and proteins in the skin. It is known that trypanosomes are able to use lipids from their surrounding environment to synthesis their own phospholipids for their membrane [49,50]. This increase in phosphate groups could thus be the result of parasites migrating into the skin or be the result of phospholipids from the host being made available for the growth of the parasite. It is possible that the abundant VSG coat proteins from the surface of the parasites, or soluble VSG released by the parasites to evade the immune response from the host [51], are detected. The alteration in the lipids/proteins composition in the skin was also supported by a shift observed in the C-H region of the spectrum in Fig 2. This region is usually characteristic of proteins and lipids. It was not possible to observe it *in situ* as the portable instrument used has a fixed grating, unable to measure vibrations at high wavenumbers.

The main energy source for trypanosome parasites present in the bloodstream is glucose [52,53], but it has been shown that parasites are also able to switch to certain lipids, such as low density lipoproteins (LDL) and high density lipoproteins (HDL) under particular conditions [49,54,55]. Moreover, in a recent study Trindade *et al*. described this change of energy source towards lipids and fatty acids present in the skin by trypanosomes that associate with the adipose tissue [50]. The presence of parasites in the skin with such metabolic adaptations could lead to an up-regulation of the release of lipids from the adipocyte to promote their proliferation and may explain the spectral variations in Raman peaks compared to uninfected tissue.

Interestingly, there was a Raman peak specific to the infection at 1674 cm^-1^ observed in some, but not all Raman spectra. This peak, assigned to an Amide I vibration, could relate to the alteration of the protein conformation in the skin due to the presence of the parasite. However, it was also described in a different study to be related to a specific lipid; cholesterol [46]. A relationship between cholesterol, LDL and HDL concentrations with trypanosomiasis have been described and showed that their concentration was reduced in the serum of animals infected with *T*. *b*. *brucei* [56,57]. This possible increased presence of cholesterol, and lipids in general, was intriguing as similar findings were obtained in mice infected with the related, intracellular parasite *Trypanosoma cruzi*, which causes Chagas disease in Latin America. Cholesterol levels were increased in the heart, pancreas and mostly in white adipose tissue during an infection by *T*. *cruzi* [58]. Although both parasites possess a different life cycle and mechanism of infection, it offers a possible alternative metabolism route by which *T*. *b*. *brucei* in skin and adipose tissue could also lead to an increased liberation of lipids such as cholesterol in the skin to promote their proliferation.

It was also noticed that porphyrin, associated with a Raman peak at 670 cm^-1^, was detected in a higher concentration than in uninfected mice and fluctuated approximately in line with parasite numbers in the skin and blood. Porphyrins are a critical structural component of haemoglobin, therefore it is possible that extravasation of parasites from blood vessels into the skin could result in increased vascular leakage of blood into the skin and might indicate a specific process of *T*. *b*. *brucei* pathology that could be used to formally detect the infection.

Biological changes observed in the Raman spectra might be the result of the detection of the parasite itself within the skin, its influence on the surrounding matrix as well as a potential host reaction to its presence. Moreover, it could be the result of an up-regulation caused by the release of certain biomolecules such as lipids by the host or the release by the parasite itself of molecules such as VSG or pyruvate derivatives in the host [51]. Indeed, it was shown in other studies that trypanosomes were able to use amino acids to release a pyruvate derivative, indolepyruvate, to modulate the host immune response [59]. Additional analytical supporting methods might be required to confirm the biological interpretation of the changes observed here in *T*. *brucei* skin, but this study demonstrates primarily that Raman spectroscopy is a viable option to detect *T*. *brucei* infection by targeting the skin. These additional analytical techniques could help identifying specific biological changes/biomarkers of the infection. It was observed during the *in situ* Raman spectroscopy time course of *T*. *b*. *brucei*-infected mice, that a spectral change could be distinguished in the skin up to 4 days before trypanosomes could be detectable by sensitive molecular methods, such as qPCR that detect trypanosome DNA. This early sensitivity could be attributed to the detection of host or parasite biomarkers such as released soluble VSGs, before the parasite is established within the skin, or to a lower threshold of detection for parasite numbers that are already present in the skin. This would be extremely beneficial in the field to identify latent infections, which are currently the limiting factor to eliminate the disease [18–22]. These patients exhibit very low parasitaemia and are difficult to confirm positively by optical microscopy. Parasites from such patients were recently demonstrated in skin punches from HAT and latent infected individuals during a disease screening study in Guinea, even in the absence of detectable parasitemia [24]. However, punch biopsies are an invasive procedure requiring trained medical personnel and equipment, wound healing, and access to a histopathological and/or molecular laboratory for analysis. Additionally, individuals are often reticent to undergo such procedure to confirm the infection due to the discomfort and inconvenience it causes. Punch biopsies are thus not currently a routine diagnostic method for HAT infection.

Raman could offer an alternative method of detecting the presence of trypanosomes in the host and thus offering a less invasive and easily transposable technique for use in the current field screening. Furthermore, if the presence of specific biomarkers is confirmed, it would open up the analysis of different biological samples with Raman such as the current blood/plasma analysis already being performed in the field.

Similarly, work on the use of Raman spectroscopy to detect the infection in mouse skin should be expanded to include further trypanosome strains as well as other common infectious diseases present in HAT-endemic regions of Africa. Here, two well characterised *T*. *b*. *brucei* strains (STIB247 and GVR35) have been used in an established animal model for studying *T*. *brucei* with predictable extravasation timepoints into the skin. Although Raman was able to differentiate both strain infections compared to uninfected controls (Fig 4), there remained a slight difference between the two strains on the PCA score plot, suggesting a possible biological variation in their interaction with the host. It was also observed that the Raman signal from trypanosome infected mice was markedly different from *P*. *berghei* infected mice, suggesting a potential infection specificity in the measurement with Raman spectroscopy. Further studies on a larger panel of trypanosome strains and infections with an increased sample size would confirm its specificity and strengthen the relevance of the use of Raman spectroscopy as a diagnostic tool.

This study demonstrates a proof of concept in using Raman spectroscopy to detect a *T*. *brucei* infection in a mammalian host by targeting the skin. It was further demonstrated that infection could be detected *in situ* by using a portable instrument, and at an earlier timepoint in infection than by a qPCR method. As we approach the WHO target for elimination of HAT, this label-free and non-invasive analytical technique could be of beneficial for the detection of *T*. *b*. *gambiense* in human patients in the field, particularly latent infections with skin-dwelling parasites but no detectable parasitemia that may be missed by current screening methods.

## Conclusion

This study demonstrated that Raman spectroscopy is a viable tool for the detection of *T*. *brucei* infection in a laboratory mouse model. Further analysis will be conducted on live infected mice tracking the infection with a portable Raman instrument, which will give more indication on its potential use in the field. We have reported biological changes in the Raman spectra of *T*. *b*. *brucei* infected skin and attempted to give an indication of the origin of these variations. While developing this new diagnostic technique, efforts should be directed towards resolving the underlying biology of *T*. *brucei* infection in the host skin which may help further increase the sensitivity and specificity of the detection approach and provide further insight into parasite-host interaction. The skin as a target of measurement offers a new possibility for the detection of the disease that may in the future help with the identification of missed cases in the field. The validation and implementation of this technique in the field may contribute to the elimination goal for *gambiense*-HAT set by WHO to 2030 with potential to expand to include Animal African Trypanosomiasis which is a large economic burden for the rural population in Africa.

## Materials and methods

### Ethics statement

All animal experiments were approved by the University of Glasgow Ethical Review Committee and performed in accordance with the Home Office guidelines, UK Animals (Scientific Procedures) Act, 1986 and EU directive 2010/63/EU. All experiments were conducted under SAPO regulations and project licence number: 200120043.

### *Trypanosoma brucei brucei* bloodstream culture

*Trypanosoma brucei brucei* bloodstream form STIB247 cell cultures were cultivated following standard protocols. In brief the trypanosomes were maintained *in vitro* in modified HMI9 medium [60], supplemented by 1.5 mM glucose, 1 mM methyl cellulose, 250 μM adenosine, 150 μM guanosine and 20% (v/v) Serum Plus media supplement (Sigma-Aldrich). Cultures were maintained in vented lid flasks in a 37°C, 5% CO_2_ incubator and sub-passaged every 2–3 days to maintain a cell density of ~10^5^−10^6^ trypanosomes per mL.

### Preparation of *Trypanosoma brucei brucei* bloodstream culture for Raman spectroscopy

Bloodstream form trypanosome (STIB247) culture cells (~10^6^) were centrifuged at 1000g for 10 minutes, washed with DPBS and then fixed with 4% paraformaldehyde for 10 minutes. After washing twice with DPBS, cells were resuspended in 10uL and spread on Raman grade calcium fluoride slides (Crystran Ltd, UK) and allowed to air-dry. Prior to Raman analysis, slides were gently washed with a few drops of water deposited and removed with a 1 mL pipette.

### Raman spectroscopy instrumentation

All Raman *in vitro* and *ex vivo* experiments were performed with an Alpha 300 R Witec confocal Raman microscope (Witec, Germany) equipped with a 532 nm laser, coupled with an Andor CCD camera (DV401-BV) with a 600 l/mm grating (spectral resolution of 3 cm^-1^), and a 785 nm laser, coupled with an Andor CCD camera (DU401-BR-DD) with a 300 l/mm grating. The microscope was equipped with a 100x (0.9 NA) lens. The spectral range used was 200–4000 cm^-1^. Before any measurements, instrument was calibrated using silica, where the Raman peak was set to 520 ± 0.5 cm^-1^, by adjusting the laser wavelength on the Witec control software toolbox (Witec, Germany).

### *Trypanosoma brucei brucei* bloodstream culture Raman imaging

Samples were analysed using a 532 nm laser, at 9 mW, a 6 s acquisition time and a spectral centre of 2500 cm^-1^. Parasites were mapped with a 0.5 μm step in x and y. Prior to the production of the Raman intensity maps, cosmic rays were removed from each spectrum using the cosmic ray removal tool for the Witec project software. Raman intensity maps were produced on the Witec project software by selecting individual Raman peaks. To obtain the Raman fingerprint of the trypanosome imaged, 20 Raman spectra were selected randomly across the trypanosome to give the best representation of the map.

### *Trypanosoma brucei brucei* parasite and mouse strains

The *T*. *b*. *brucei* strains used in this study are pleomorphic, laboratory strains that induce a chronic infection in mice. STIB247 was originally isolated from a hartebeest in the Serengeti in 1971 [61]. GVR35 was originally isolated in the Serengeti from a wildebeest in 1966. BALB/c and C57BL/6J mice were used as models for chronic *T*. *brucei* infection [62].

### *Trypanosoma brucei brucei* mouse infections

Eight week old female BALB/c or C57Black/6J mice (Harlan, UK) was inoculated by intra-peritoneal (IP) injection with 10^4^ parasites of strain STIB247 or GVR35. Parasitaemia was monitored by regular sampling from the superficial tail vein and examined using phase microscopy and the rapid “matching” method [63]. Uninfected mice of the same strain, sex and age served as uninfected controls. Excised skin sections from euthanised mice were preserved by fixation in 10% neutral buffered formalin or freezing at -80°C, as appropriate to the experiment.

For *in situ* analysis, mice (BALB/c or C57/Black/6J) were euthanised at a defined time point during the infection, excess of hair were removed from the abdominal region and Raman measurement were taken directly onto this region. These mice were randomly assigned to a specific experimental time point at the beginning of the infection by tail marking, without blinding method.

### Preparation of trypanosome infected and uninfected murine skin samples for *ex vivo* Raman spectroscopy

Previously validated archival skin from a BALB/c mouse with a moderate STIB247 extravascular infection and an uninfected control mouse were used to prepare *ex vivo* murine skin samples [23]. Briefly, at day 15 of infection the mice were euthanised by approved Schedule 1 method and 2 cm^2^ skin samples were removed from the abdominal area for both infected and uninfected mice. Skin samples were fixed in 10% neutral buffered formalin for 48 hours before being trimmed and processed into paraffin blocks. Paraffin embedded formalin fixed skin samples were cut into 2.5 microns sections and placed on CaF_2_ slides (Crystran Ltd, UK). Prior to Raman analysis, skin samples were dewaxed to remove the paraffin. Samples were placed in a bath of xylene (Sigma-Aldrich, UK) for 15 min. This step was repeated twice. Samples were then washed successively with 90% ethanol (v/v), 70% ethanol (v/v) and then water, for a period of 5 min each. Wash steps with water were repeated three times.

### *Plasmodium berghei* mouse infection and sample preparation for *ex vivo* analysis

One TO mouse was inoculated by intra-venous (IV) injection with 1x10^6^ schizonts of *P*. *berghei* ANKA strain [64], with one uninfected TO mouse serving as a control. At day 3 post infection, the infected TO mouse was euthanised by approved Schedule 1 method, excess hair from its lower ventral region was removed using Nair hair removal cream, and 4 mm^2^ skin sample was removed from this region. The skin was fixed in 10% neutral buffered formalin for 24h before being processed and embedded in paraffin blocks. Paraffin embedded formalin fixed skin were cut and placed onto a CaF_2_ slides (Crystran Ltd, UK). Uninfected control mouse skin sample was processed identically.

Prior to Raman analysis, skin samples were dewaxed to remove the paraffin wax. Samples were placed in a bath of xylene (Sigma-Aldrich, UK) for 15 min. This step was repeated twice. Samples were then washed successively with 90% ethanol (v/v), 70% ethanol (v/v) and then water, for a period of 5 min each. Wash steps with water was repeated three times, air dried and analysed by Raman spectroscopy.

### Raman mapping and PCA analysis of *ex vivo* uninfected and *T*. *b*. *brucei* infected mice skin samples

Prepared *ex-vivo* skin samples were mapped in square dimensions (50 x 50 μm) using a 2 μm step in x and y, for a total of 625 spectra, using a 532 nm laser, 12 mW, with a 1 s acquisition time and a spectral centre of 2450 cm^-1^. Seven maps for each skin samples were imported into Matlab (Mathworks, UK) and pre-processed as follows: cosmic rays were removed using a length factor 5, spectra were smoothed with Savitzky-Golay smoothing method with a polynomial order of 2 and spectral width of 9, the spectral window was reduced to 500–3600 cm^-1^ and data were baseline corrected with a smoothing parameter of 10^4^ and asymmetry parameter of 0.01. Each map (625 spectra for each map) was then averaged into a single Raman spectrum for both samples, a total of seven Raman spectra for each uninfected and infected skin and normalised individually to the highest peak intensity. Data set was mean centred before being analysed using PCA algorithm.

For the Raman fingerprint comparison between *ex vivo* uninfected and infected skin each data sets from uninfected and infected skin (seven Raman spectra for each) had their spectral window divided in two separate sections: 550–1800 and 2550–3600 cm^-1^. Spectra from each sample were averaged into a single Raman fingerprint, representing one spectrum for each state of the skin, and were normalised separately to the highest peak intensity before comparison.

The standard deviation for the seven spectra from each uninfected and infected sample were plotted along with their respective averaged Raman spectrum prior the normalisation step (S3 Fig).

### Raman mapping of *ex vivo* skin from uninfected and *Plasmodium berghei* infected mice and comparison with *T*. *b*. *brucei* infected mice

Both *ex vivo* skin samples from uninfected and *Plasmodium berghei* infected mice were analysed using the same method described previously for *ex vivo skin samples from T*. *b*. *brucei* infected mice.

The seven maps obtained from each sample were also pre-processed using the same method and each map was averaged into a single Raman spectrum for both samples, obtaining a total of seven Raman spectra for each uninfected and infected sample. Their spectral window was then divided into two separate sections: 550–1800 and 2550–3600 cm^-1^ and the seven Raman spectra were subsequently averaged into a single Raman fingerprint for each state of the skin. Finally, both Raman spectrum for uninfected and infected skin were normalised to the highest peak intensity.

The Raman signal obtained for both infections (*T*. *b*. *brucei* and *Plasmodium berghei*) were compared following this method: each infected normalised Raman spectrum were subtracted by their respective uninfected normalised Raman spectrum, providing a Raman signal specific to the biological changes induced in the skin by each infection. Both Raman pattern were plotted against each other for comparison.

### Raman fingerprint comparison between *T*. *b*. *brucei*, uninfected and infected skin sample

Five *T*. *b*. *brucei* were mapped for this analysis. From each map, 20 Raman spectra were randomly selected to represent the signal obtained from each African trypanosomes analysed (total of 100 Raman spectra), and data were imported in Matlab (Mathworks, UK). The data set was pre-processed as follow: cosmic rays were removed using a length factor 5, spectra were smoothed with Savitzky-Golay smoothing method with a polynomial order of 2 and spectral width of 9, the spectral window was split between 550–1800 cm^-1^ and 2550–3600 cm^-1^, both data were baseline corrected with a smoothing parameter of 10^4^ and asymmetry parameter of 0.01. The data set was then averaged into a single Raman spectrum for each map, to obtain five Raman fingerprint for each map.

The five Raman spectra, for each spectral window, were then averaged into a single Raman spectrum, normalised to the highest peak intensity and plotted against the spectra obtained previously for the uninfected and infected skin for comparison.

The standard deviation for the five Raman spectra was plotted along with its respective single Raman spectrum prior the normalisation step (S5 Fig).

### Raman analysis on mouse in situ

All Raman analysis were performed using a Snowy Range benchtop (Snowy Range instruments, UK), model sierra 2.0, 785 nm laser (100 mW). The spectral range of the measurement was 200–2000 cm^-1^ with a spectral resolution of 4 cm^-1^. All measurements were performed using the bottom aperture of the instrument.

The instrument was elevated from the bench, and a plate holder was placed underneath the aperture, which could regulate the distance between the sample and the aperture. Prior to any measurements, the instrument was calibrated with a silicon slide, which provided the optimal focus point of the laser. This distance was measured and applied during the analysis of mice in order to keep the working distance identical between experiments.

Excess hair was removed from euthanised mice (BALB/C or C57/Black6J) infected with *T*. *b*. *brucei* STIB247 or GVR35, or matched uninfected control using a hair removal cream (Veet) and following the manufacturer’s instructions, followed by thoroughly washing of the area with water.

Mice were then placed on the plate holder and the working distance was defined. Raman spectra were obtained with three acquisitions, at 30 s (for Fig 5) and 60 s (for Fig 4) acquisition time and 45 mW, and ten (Fig 4) or five (Fig 5) replicates were obtained across the skin depending on the experiment. The Orbital Raster Scan (ORS) was not used during the experiments.

### Data processing and analysis for in situ experiments

After Raman experiments, data were saved as SPC files and imported in Matlab 2016a (Mathworks, UK). The three acquisitions for each single point measurement were averaged into a single Raman spectrum. Each spectrum was smoothed with Savitzky-Golay smoothing method with a polynomial order of 3 and spectral width of 15, the spectral window was reduced to 599–1774 cm^-1^ and data were baseline corrected with a smoothing parameter of 10^5^ and asymmetry parameter of 0.01.

The Raman spectra replicates from each mouse were then averaged into a single Raman spectrum representative of the mouse. Finally, each spectrum was normalised to the highest peak intensity and plotted on the same figure for comparison.

### Semi-quantitative evaluation of the parasite burden and inflammation in skin sections

Paraffin embedded skin samples were cut into 2.5 micro sections and stained for *T*. *brucei* parasites using a polyclonal rabbit antibody raised against *T*. *brucei* invariant surface glycoprotein 65 (ISG65) (M. Carrington, Cambridge, UK) using a Dako Autostainer Link 48 (Dako, Denmark) and were subsequently counterstained with Gill’s Haematoxylin. The extent of inflammatory cell infiltration in skin sections was assessed in haematoxylin and eosin stained sections. Stained slides were assessed by a veterinary pathologist blinded to infection status and experimental procedures.

Parasite burden was assessed for both intravascular location (parasites within the lumen of dermal or subcutaneous small to medium-sized vessels) and extravascular location (parasites outside blood vessels, scattered in the connective tissue of the dermis or in the subcutis) and was evaluated in 10 randomly selected high-power fields at x40 magnification for each sample. A semi-quantitative ordinal score was used to grade the trypanosomes burden in the skin, where 0 showed no presence of parasites, 1 showed low number of parasites (<20 trypanosomes), 2 showed a moderate number of parasites (20–50 trypanosomes) and 3 showed large numbers of parasites (>50). An average parasite burden score per field of view was calculated for each sample.

Inflammation was assessed with a similar semi-quantitative scoring system, graded on a 0–3 scale: 0 (absent or only rare leukocytes present), 1 (mild inflammation–low numbers of mixed inflammatory cells present), 2 (moderate–moderate numbers of mixed inflammatory cells), and 3 (large numbers of inflammatory cells). The average of 10 randomly selected high-power fields at x20 magnification for each sample determined the inflammatory score.

### DNA extraction and Quantitative Polymerase Chain Reaction (qPCR) of murine skin tissue

Genomic DNA (gDNA) was extracted from 25–30 mg skin tissue preserved at -80oC. After defrosting on ice, skin was finely chopped with scissors, and disrupted for 5 minutes in 180uL ATL buffer (Qiagen) using a Qiagen Tissuelyser at 50Hz with a ceramic bead (MPBio). Disrupted tissues was incubated at 56oC with 2mg/ml proteinase K (Invitrogen) overnight and DNA extracted from digested tissue using the Qiagen DNeasy Blood and Tissue Kit (Qiagen, Manchester (UK)). The resulting gDNA was quantified using a Qubit Fluorimeter (Thermofisher Scientific). and diluted to 4 ng/μl.

Trypanosome load in the skin was determined using Taqman real-time PCR, using primers and probe specifically designed to detect the trypanosome *Pfr2* gene as previously reported [48]. Reactions were performed in a 25 μl reaction mix comprising 1xTaqman Brilliant II master mix (Agilent, Stockport, UK), 0.05 pmol/μl forward primer (CCAACCGTGTGTTTCCTCCT), 0.05 pmol/ μl reverse primer (GAAAAGGTGTCAAACTACTGCCG), 0.1 pmol/μl probe (FAM- CTTGTCTTCTCCTTTTTTGTCTCTTTCCCCCT-TAMRA) (Eurofins MWG Operon, Eurofins Genomics, Germany) and 20 ng template DNA.

A standard curve was constructed using a serial dilution range: 1x 10^6^ to 1x 10^1^ copies of pCR 2.1 vector containing the cloned *Pfr2* target sequence (Eurofins MWG Operon, Eurofins Genomics, Germany). The amplification was performed on an Mx Pro 3005 (Agilent, USA) with a thermal profile of 95°C for 10 minutes followed by 45 cycles of 95°C for 15 seconds, 60°C for 1 minute and 72°C for 1 second. The trypanosome load within the skin sample was calculated from the standard curve using the MxPro qPCR software (Agilent, USA). *Pfr2* copy numbers were divided by the gene copy number in the *T*. *b*. *brucei* genome (four copies) to calculate the approximate number of trypanosomes present in the sample. Where appropriate, gene values are normalised to tissue weight based on the representative DNA yield for skin tissue for the DNA extraction method (952 ug DNA per mg skin tissue).

### Statistical analysis

Principal component analysis was performed with Matlab 2016a (Mathworks, UK) and an in-house statistical toolbox.

## Supporting information

S1 Fig Raman spectrum of *T. brucei* and some examples of its associated false colour image. Data obtained with a 532 nm laser, 6 s acquisition time and 100x lens. Representative Raman spectrum of the parasite taken from a single point on the parasite, as shown in Fig 1 and false coloured image associated with other Raman peaks related to the parasite with their respective intensity bar, which assign a colour gradient from the lowest (black) to the highest intensity (white).
(TIF)

S2 Fig Loading Spectra of the principal component associated to the PCA scores plot of *ex vivo* murine BALB/C skin analysis. The loading spectra for principal component 1 (A) and 2 (B) are related to the PCA performed on the ex vivo murine data shown in Fig 2.
(TIF)

S3 Fig Averaged Raman spectra for *T*. *b*. *brucei* infected (red) and uninfected (blue) BALB/C mice *ex vivo* skin on CaF_2_ slides and their respective standard deviation. Raman spectra are separated in two spectral windows: 550–1800 cm^-1^ (A) and 2550–3600 cm^-1^ (B) and were described in Fig 2.
(TIF)

S4 Fig Raman spectral comparison between *T*. *b*. *brucei* infected (red) and *P*. *berghei* infected (purple) murine *ex vivo* skin sample on CaF_2_ slides. Each infected spectra had their respective uninfected skin Raman spectra subtracted to obtain a Raman signal that was specifically induced by the infection (*P. berghei* or *T*. *b*. *brucei*) and are show in two separate spectral windows: 550–1800 cm^-1^ (A) and 2550–3600 cm^-1^ (B). The biggest spectral difference between the two infections is highlighted with black arrows in both sections for the *T*. *b*. *brucei* infected Raman spectrum.
(TIF)

S5 Fig Averaged Raman spectrum for pure *T*. *b*. *brucei* on a CaF_2_ and its standard deviation. The spectrum is separated in two spectral windows: 550–1800 cm^-1^ (A) and 2550–3600 cm^-1^ (B) and was described in Fig 3.
(TIF)

S6 Fig Picture of the portable Raman set up used during the *in situ* experiments. The Raman instrument is placed on an elevated surface and the laser is designed to irradiate a sample placed underneath the instrument as shown with the arrow.
(TIF)

S7 Fig Loading spectra of the principal component associated to the PCA scores plot of *in situ* murine (BALB/C) skin analysis. The loading spectra for principal component 1 (A) and 2 (B) are related to the PCA performed on the in situ murine data shown in Fig 4.
(TIF)

S8 Fig Images of anti-ISG65 stained samples for uninfected and trypanosome-infected skin samples taken at a magnification of x60 and x80. Histological sections of skin from uninfected and *T*. *b*. *brucei* GVR35 infected BALB/C mice stained with trypanosome-specific anti-ISG65 antibody (brown), counterstained with Gill’s Haematoxylin stain (blue).
(TIF)

S9 Fig Table of raw data scores made for the semi-quantitative evaluation of parasites burden in the skin. Trypanosomes are identified by immunohistochemical staining with the trypanosome-specific anti-ISG65 antibody. Presence of trypanosomes was assessed in 10 high power fields (HPFs) at 40x magnification and both intravascular (red) and extravascular (blue) region were scored according to the following ordinal score: 0 = no parasites detectable, 1 = low numbers of parasites (<20), 2 = moderate numbers of parasites (20–50) and 3 = large number of parasites (>50).
(TIF)

S10 Fig Table of raw data scores made for the semi-quantitative measurements of inflammation cells present in murine skin due to the infection by parasites. The extent of inflammatory cell infiltration in the skin sections was analysed by haematoxylin and eosin staining and assessed in 10 high power fields (HPFs) at 20x magnification in each sample: control group (blue), *T*. *b*. *brucei* GVR35 infection (red) and *T*. *b*. *brucei* STIB247 infection (green), and were scored according to the following semiquantitative scoring system: 0 = absent or only rare leukocytes present, 1 = mild (low numbers of mixed inflammatory cells), 2 = moderate (moderate numbers of mixed inflammatory cells) and 3 = marked (large numbers of mixed inflammatory cells)
(TIF)

S11 Fig Raman spectral comparison between BALB/C (blue) and C57/black6J (red) mice. Measurements were taken on the abdominal region with an acquisition time of 30 s and a 785 nm laser wavelength.
(TIF)

S12 Fig Loading spectra of the principal component associated to the PCA scores plot of *in situ* murine C57/black6J analysis. The loading spectra for principal component 1 (A) and 2 (B) are related to the PCA performed on the in situ murine skin data shown in Fig 5.
(TIF)

S13 Fig Raman peak intensity ratio graphs for the remaining Raman peaks that varied during the murine infection compared to uninfected control group. (TIF)
