# Supplementary figures and images for: Raman spectroscopic analysis of skin as a diagnostic tool for Human African Trypanosomiasis
Source: PLoS Pathog. 2021 Nov 15;17(11):e1010060. doi: 10.1371/journal.ppat.1010060 (PMC8629383; doi:10.1371/journal.ppat.1010060)

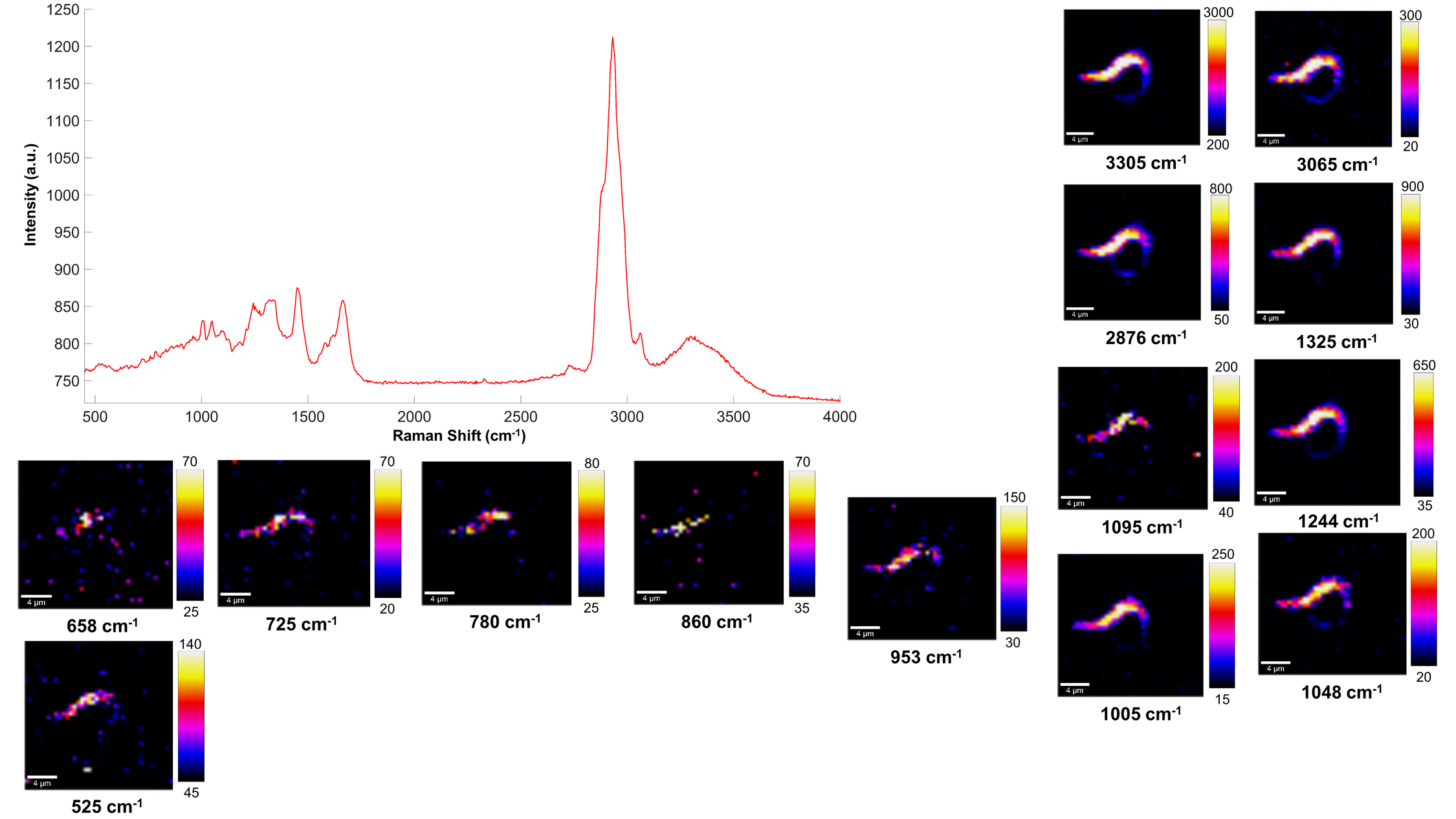

Supplement: S1 Fig — Data obtained with a 532 nm laser, 6 s acquisition time and 100x lens. Representative Raman spectrum of the parasite taken from a single point on the parasite, as shown in Fig 1 and false coloured image associated with other Raman peaks related to the parasite with their respective intensity bar, which assign a colour gradient from the lowest (black) to the highest intensity (white). (TIF) [file ppat.1010060.s001.tif]

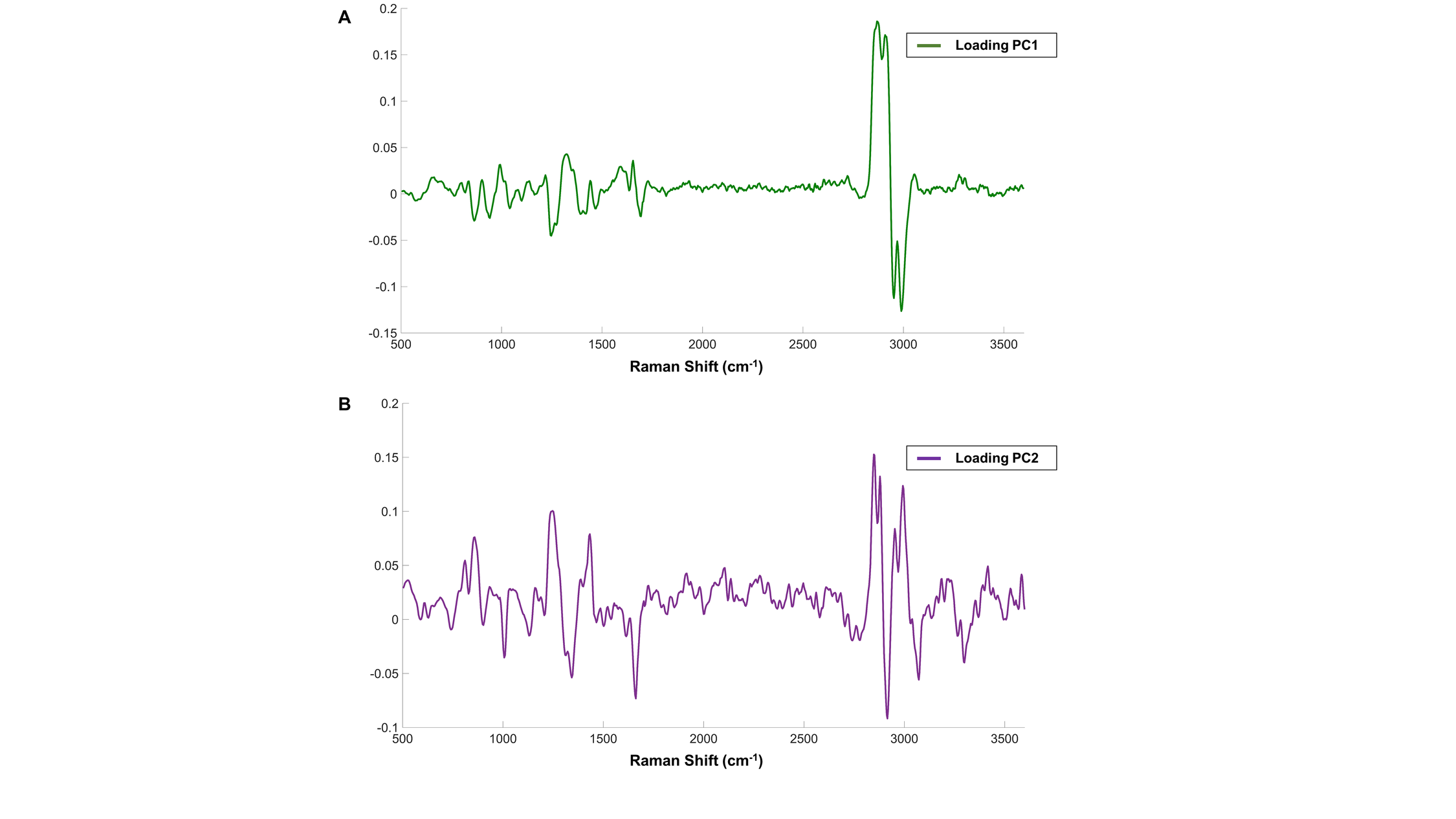

Supplement: S2 Fig — The loading spectra for principal component 1 (A) and 2 (B) are related to the PCA performed on the ex vivo murine data shown in Fig 2. (TIF) [file ppat.1010060.s002.tif]

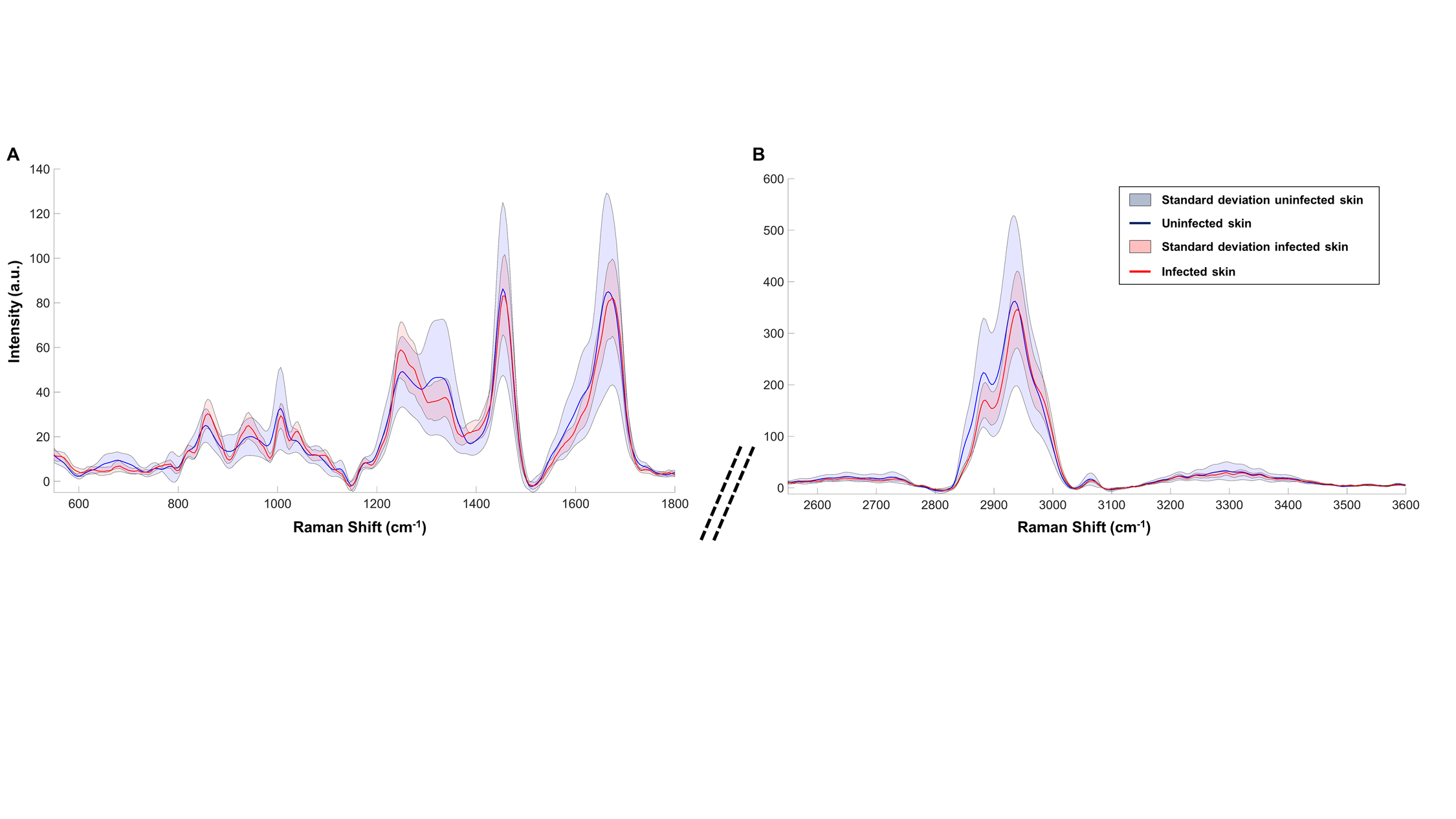

Supplement: S3 Fig — Raman spectra are separated in two spectral windows: 550–1800 cm-1 (A) and 2550–3600 cm-1 (B) and were described in Fig 2. (TIF) [file ppat.1010060.s003.tif]

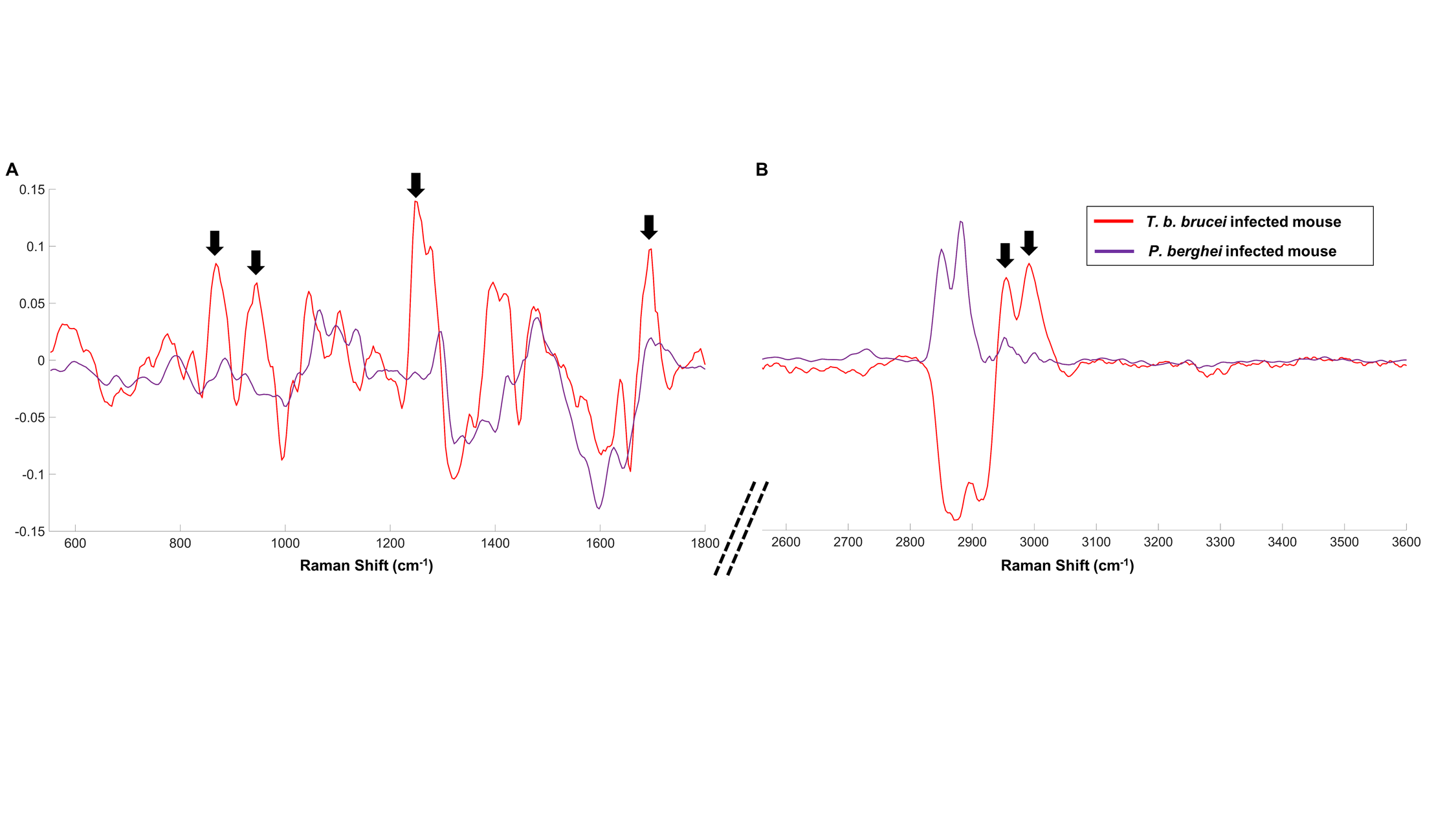

Supplement: S4 Fig — Each infected spectra had their respective uninfected skin Raman spectra subtracted to obtain a Raman signal that was specifically induced by the infection (P. berghei or T. b. brucei) and are show in two separate spectral windows: 550–1800 cm-1 (A) and 2550–3600 cm-1 (B). The biggest spectral difference between the two infections is highlighted with black arrows in both sections for the T. b. brucei infected Raman spectrum. (TIF) [file ppat.1010060.s004.tif]

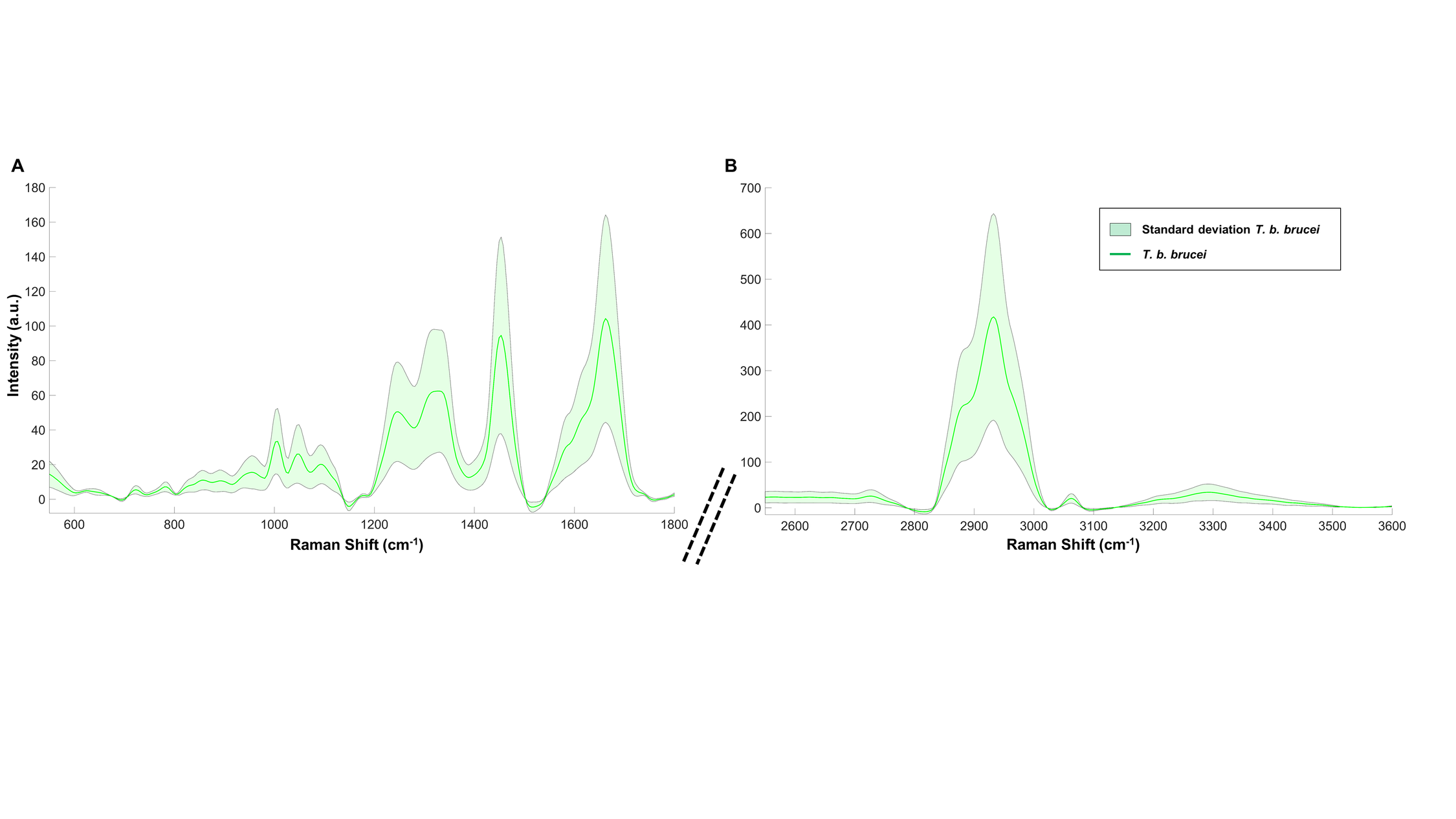

Supplement: S5 Fig — The spectrum is separated in two spectral windows: 550–1800 cm-1 (A) and 2550–3600 cm-1 (B) and was described in Fig 3. (TIF) [file ppat.1010060.s005.tif]

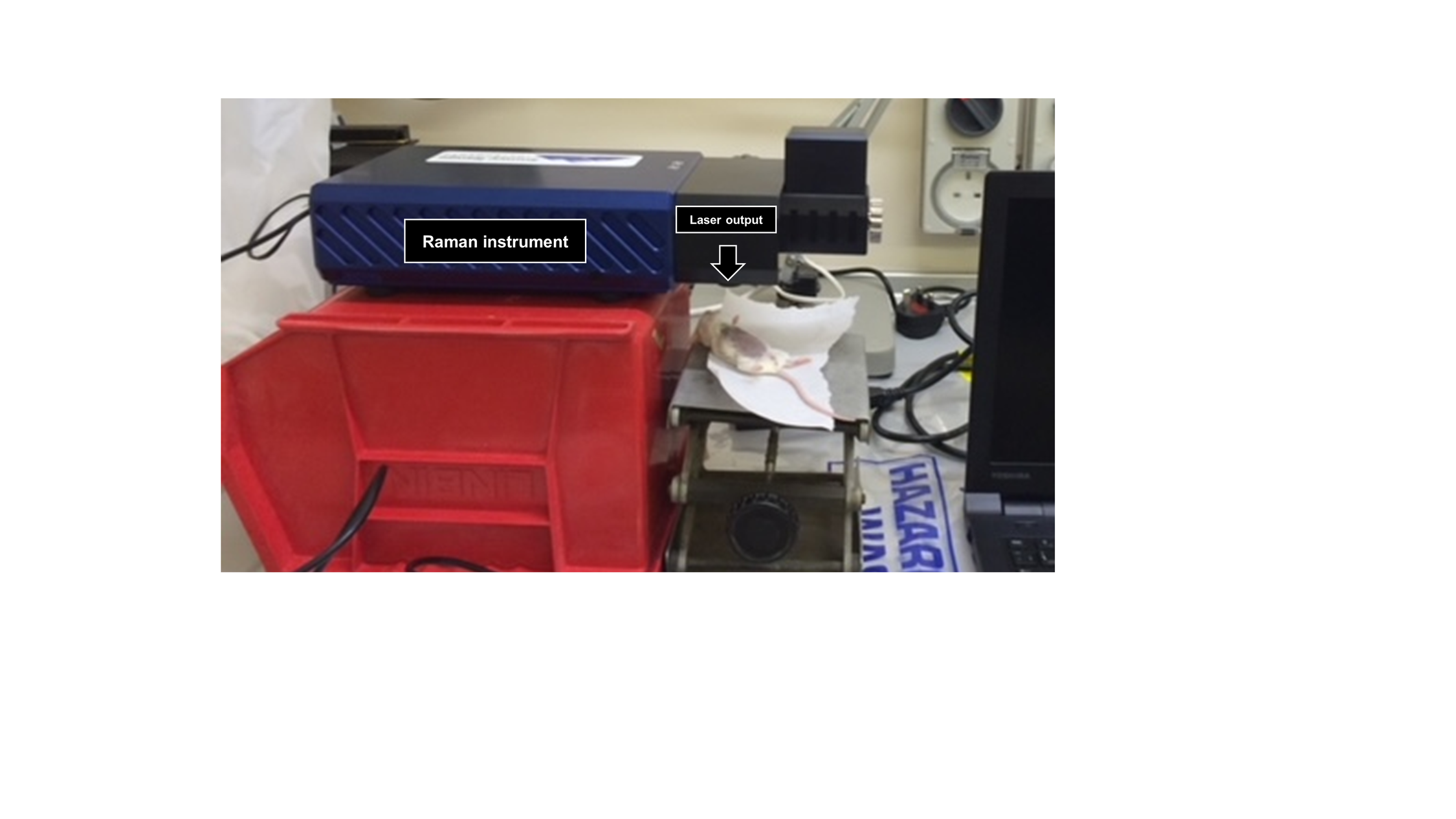

Supplement: S6 Fig — The Raman instrument is placed on an elevated surface and the laser is designed to irradiate a sample placed underneath the instrument as shown with the arrow. (TIF) [file ppat.1010060.s006.tif]

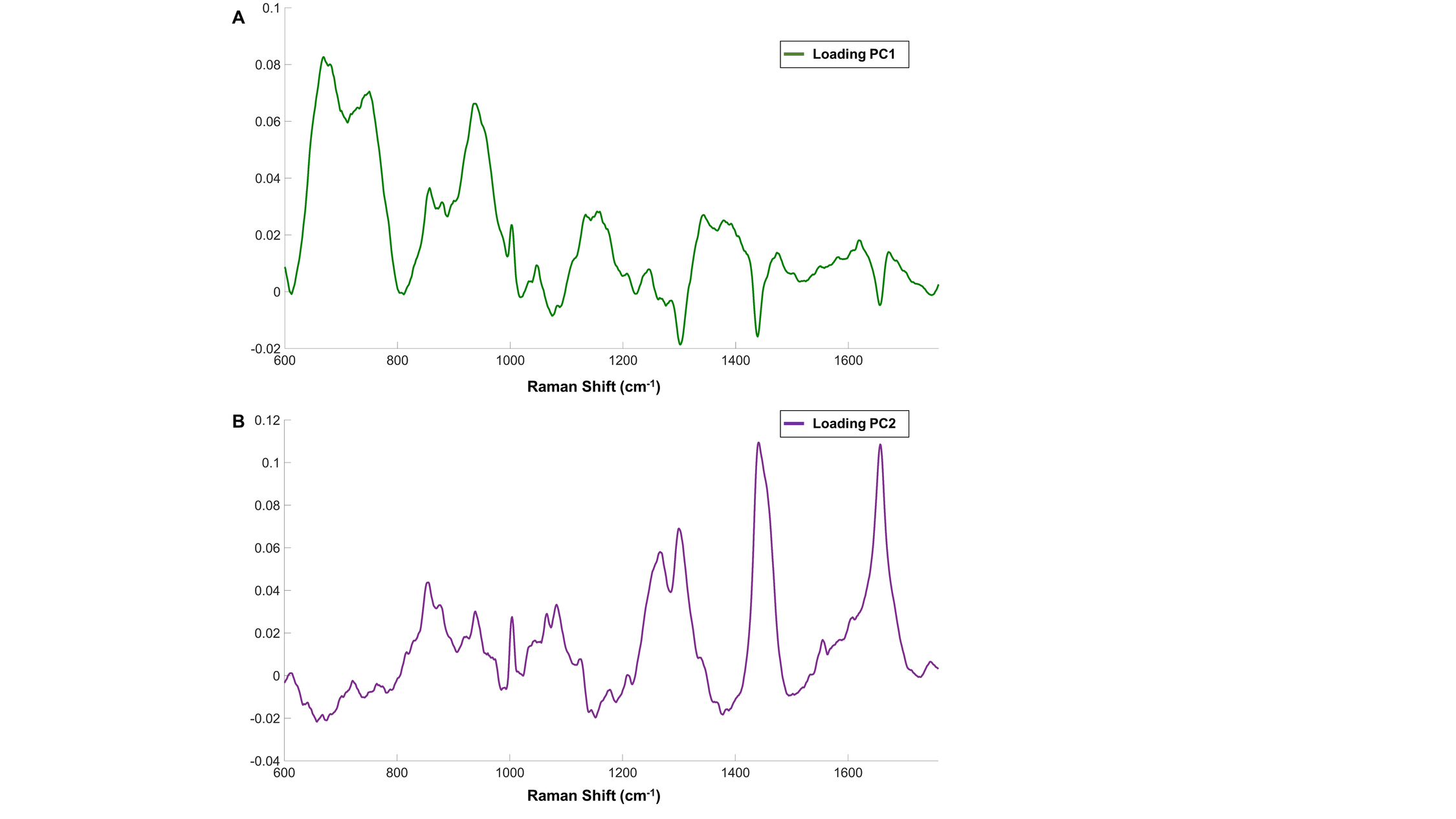

Supplement: S7 Fig — The loading spectra for principal component 1 (A) and 2 (B) are related to the PCA performed on the in situ murine data shown in Fig 4. (TIF) [file ppat.1010060.s007.tif]

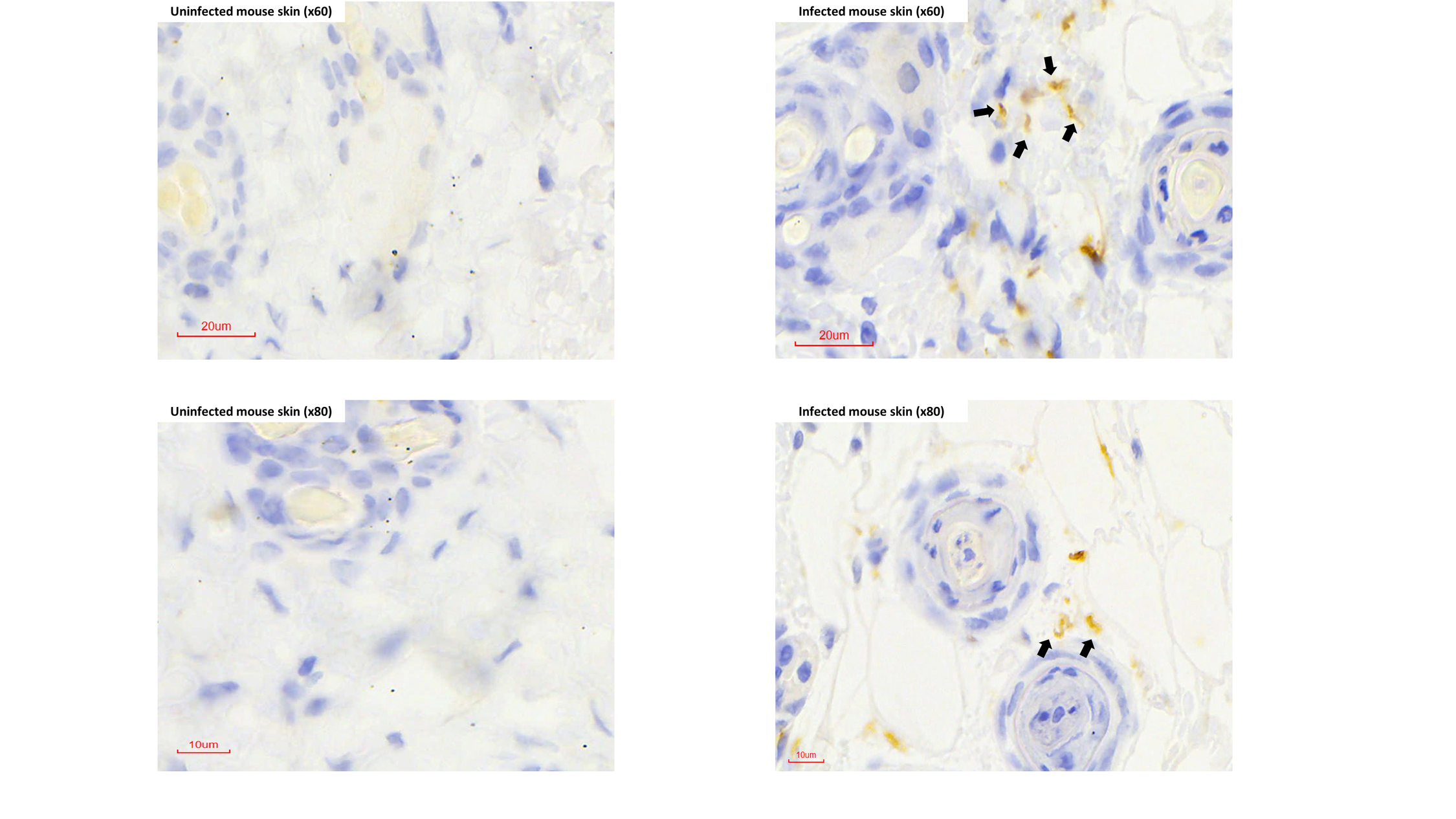

Supplement: S8 Fig — Histological sections of skin from uninfected and T. b. brucei GVR35 infected BALB/C mice stained with trypanosome-specific anti-ISG65 antibody (brown), counterstained with Gill’s Haematoxylin stain (blue). (TIF) [file ppat.1010060.s008.tif]

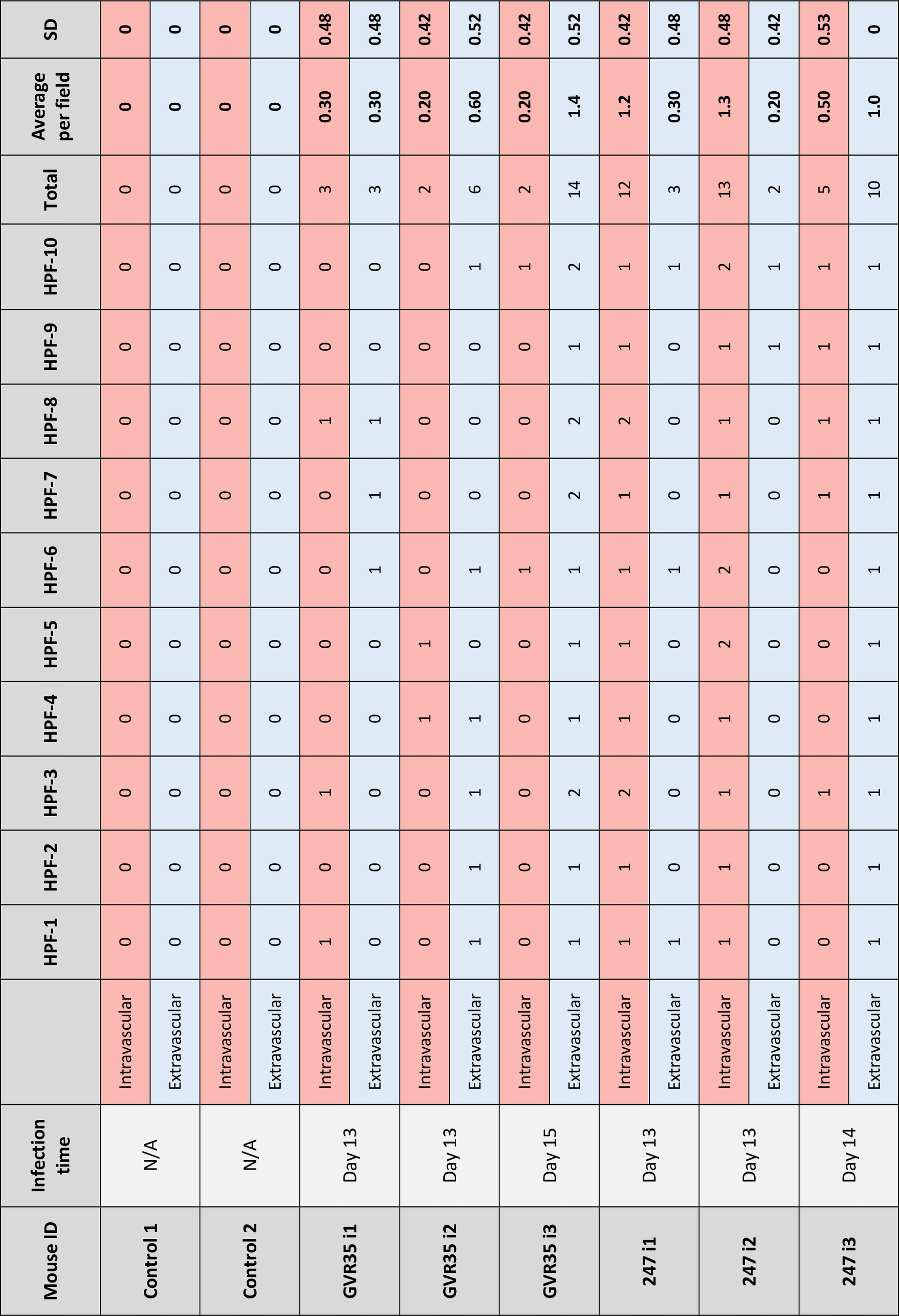

Supplement: S9 Fig — Trypanosomes are identified by immunohistochemical staining with the trypanosome-specific anti-ISG65 antibody. Presence of trypanosomes was assessed in 10 high power fields (HPFs) at 40x magnification and both intravascular (red) and extravascular (blue) region were scored according to the following ordinal score: 0 = no parasites detectable, 1 = low numbers of parasites (<20), 2 = moderate numbers of parasites (20–50) and 3 = large number of parasites (>50). (TIF) [file ppat.1010060.s009.tif]

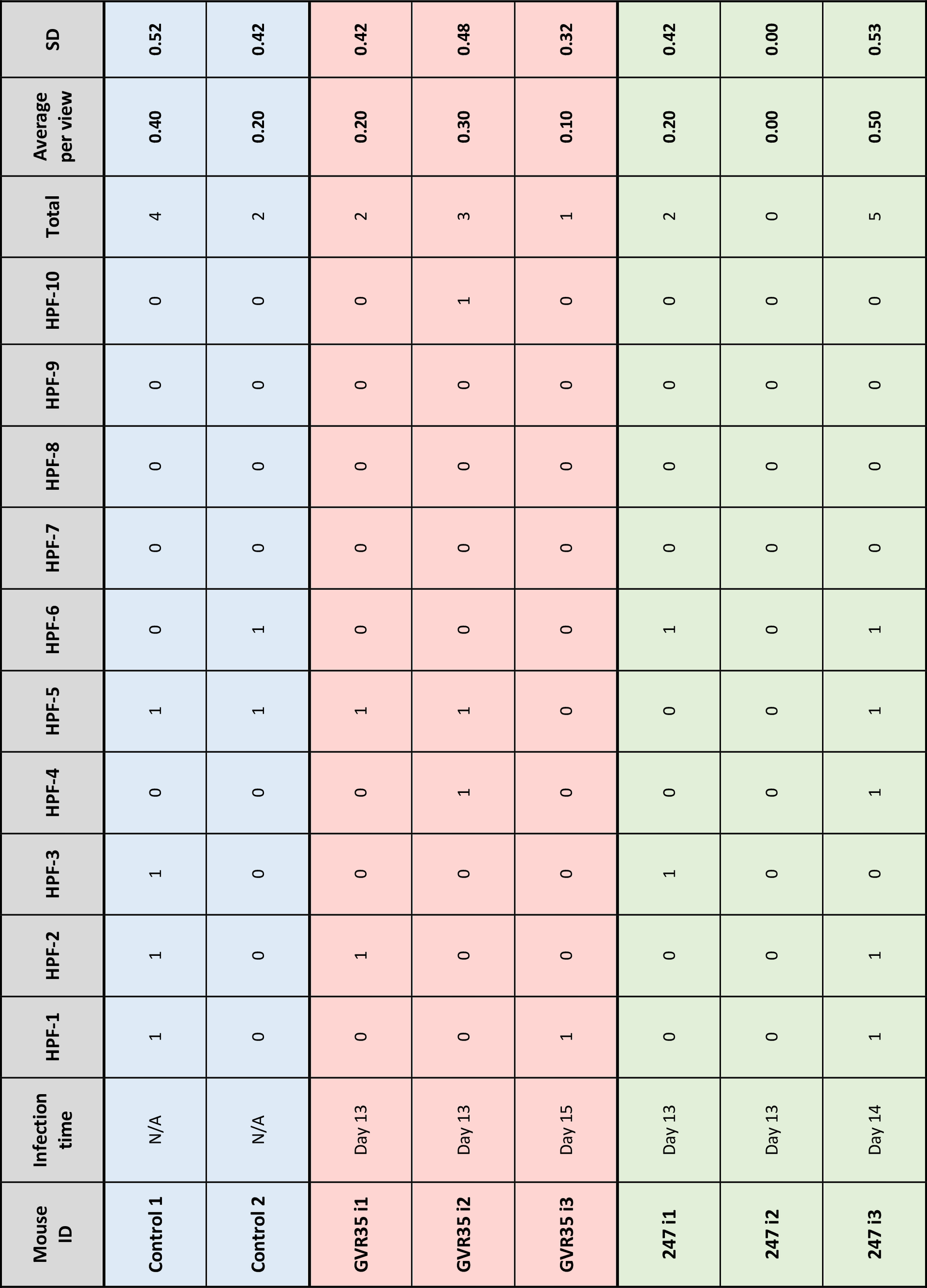

Supplement: S10 Fig — The extent of inflammatory cell infiltration in the skin sections was analysed by haematoxylin and eosin staining and assessed in 10 high power fields (HPFs) at 20x magnification in each sample: control group (blue), T. b. brucei GVR35 infection (red) and T. b. brucei STIB247 infection (green), and were scored according to the following semiquantitative scoring system: 0 = absent or only rare leukocytes present, 1 = mild (low numbers of mixed inflammatory cells), 2 = moderate (moderate numbers of mixed inflammatory cells) and 3 = marked (large numbers of mixed inflammatory cells) (TIF) [file ppat.1010060.s010.tif]

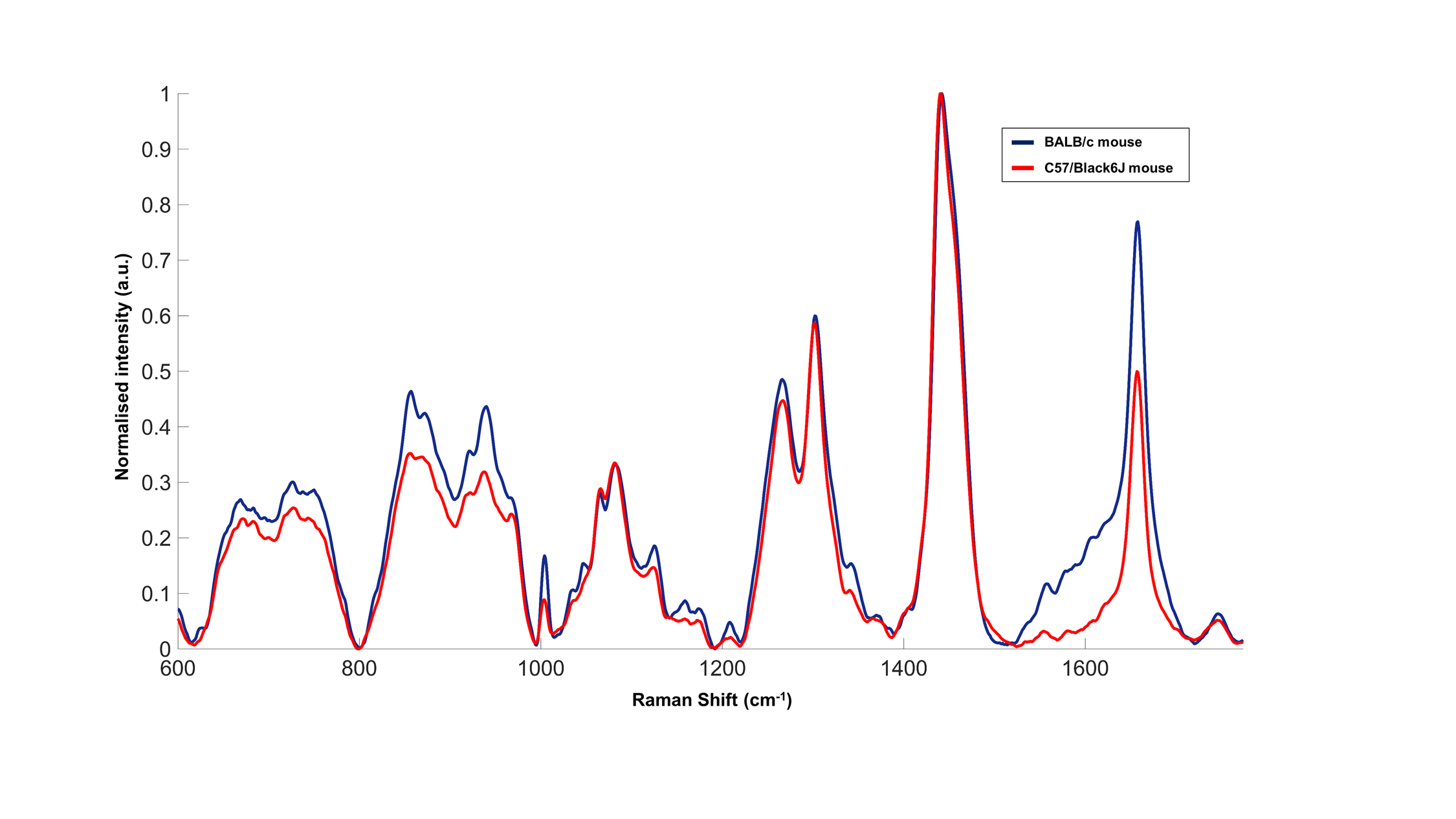

Supplement: S11 Fig — Measurements were taken on the abdominal region with an acquisition time of 30 s and a 785 nm laser wavelength. (TIF) [file ppat.1010060.s011.tif]

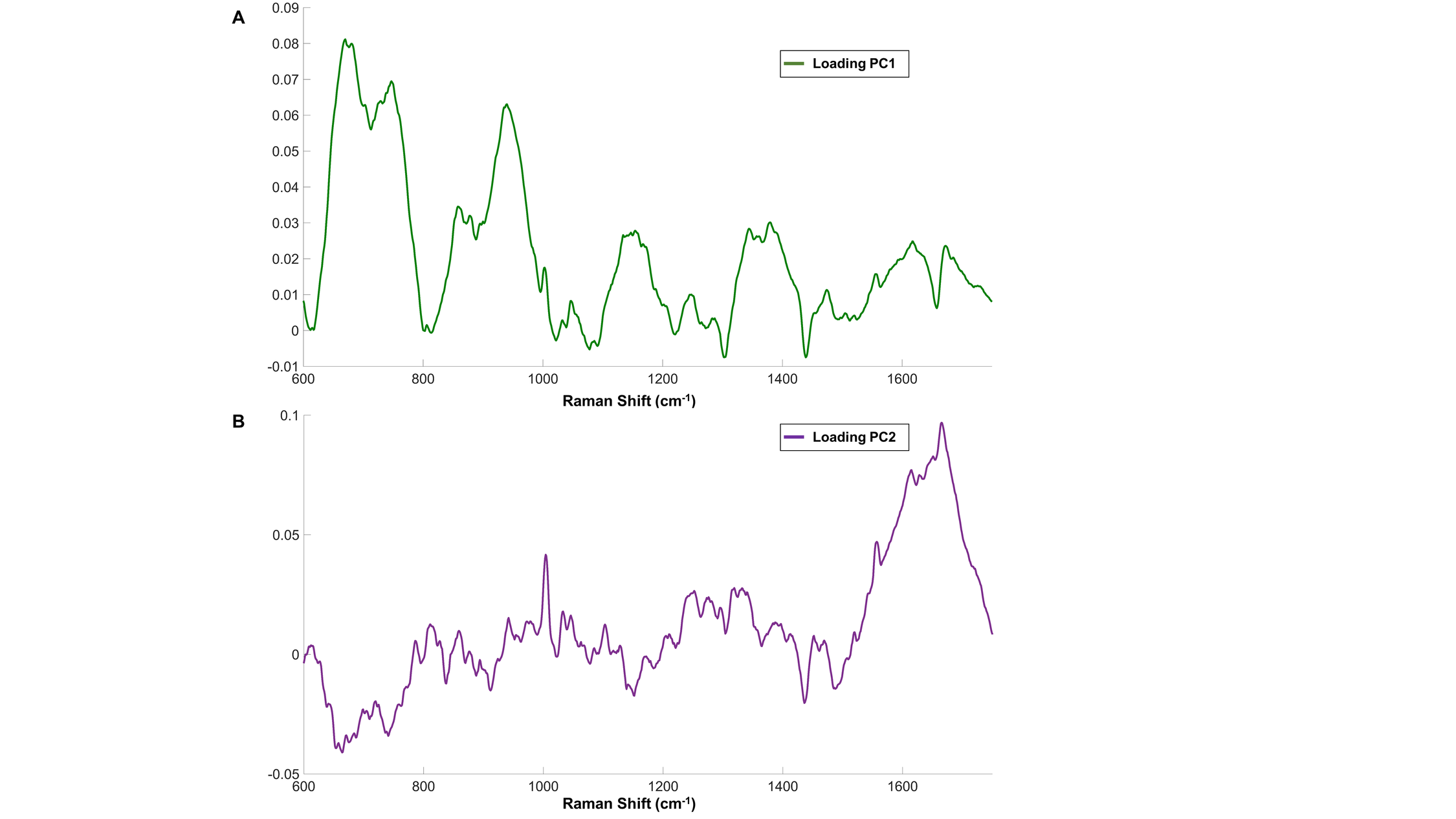

Supplement: S12 Fig — The loading spectra for principal component 1 (A) and 2 (B) are related to the PCA performed on the in situ murine skin data shown in Fig 5. (TIF) [file ppat.1010060.s012.tif]

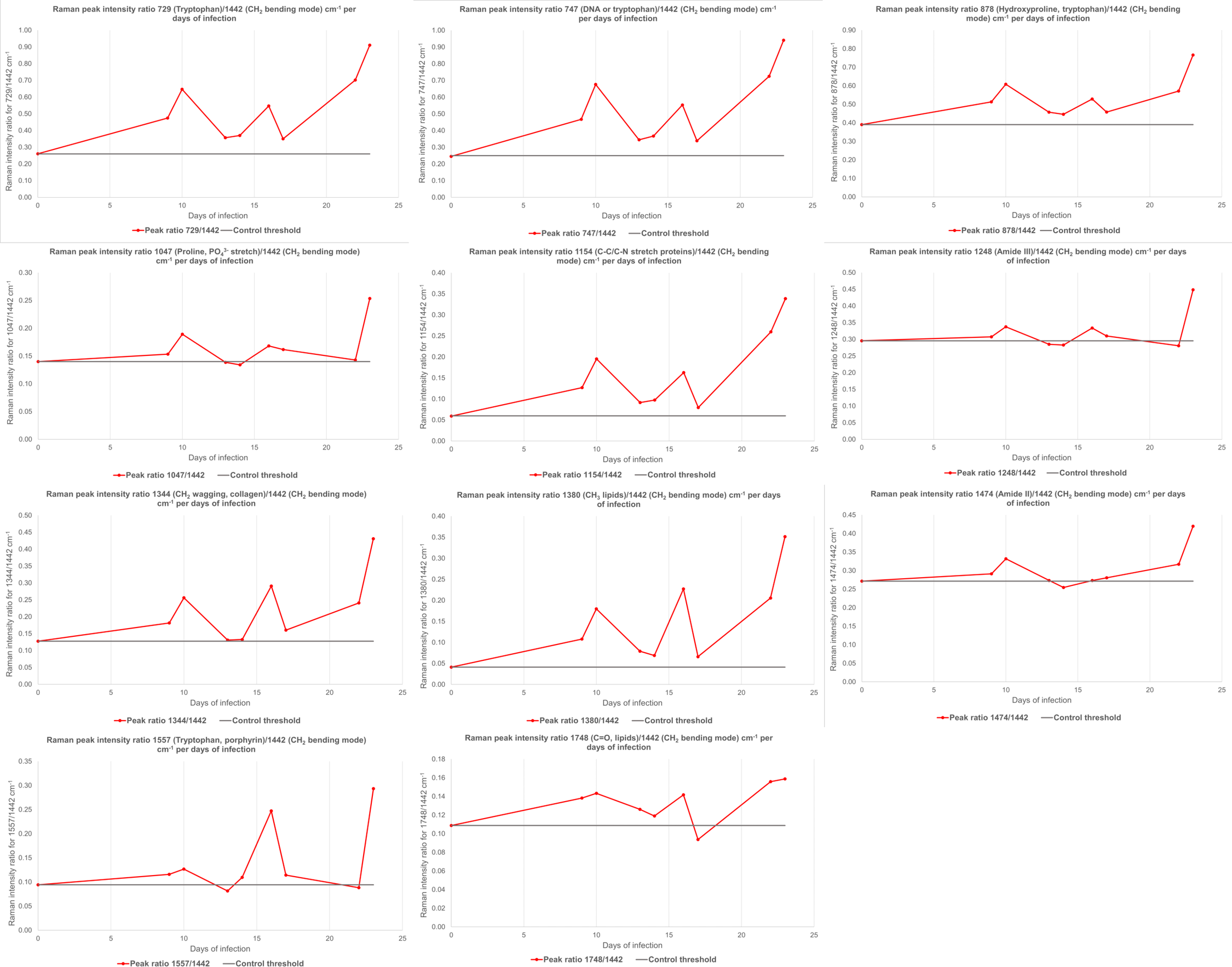

Supplement: S13 Fig — (TIF) [file ppat.1010060.s013.tif]
